# Comparative study of pathology of various organs of rhesus macaques exposed to two different doses of acute total-body radiation

**DOI:** 10.1038/s41598-026-49844-x

**Published:** 2026-04-25

**Authors:** Matthew W. Brink, Sarah A. Petrus, Alana D. Carpenter, Oluseyi O. Fatanmi, Stephen Y. Wise, Thomas M. Seed, Sang-Ho Lee, Vijay K. Singh

**Affiliations:** 1 Division of Radioprotectants, Department of Pharmacology and Molecular Therapeutics, F. Edward Hébert School of Medicine, Uniformed Services University of the Health Sciences, 4301 Jones Bridge Road, Bethesda, MD 20814-2712 USA; 2 Armed Forces Radiobiology Research Institute, Uniformed Services University of the Health Sciences, Bethesda, MD 20814 USA; 3 Tech Micro Services, 4417 Maple Avenue, Bethesda, MD 20814 USA; 4 Pathology Department, Research Services, Naval Medical Research Center, Silver Spring, MD 20910 USA

**Keywords:** Histopathology, Gamma-radiation, Nonhuman primates, Radiation-induced injury, Rhesus, Total-body irradiation, Diseases, Medical research, Physiology

## Abstract

Acute, whole-body exposure to ionizing radiation has the potential to induce widespread injury, compromising multiple organ systems and predisposing the individual to a variety of pathologies. For medical countermeasures to advance towards regulatory approval, the United States Food and Drug Administration Animal Rule requires the use of validated large animal models of acute radiation syndrome (ARS). The rhesus macaque (*Macaca mulatta*) serves as a primary model for therapeutic development due to its close genetic, anatomic, and physiological homology to humans. Characterizing the nature of ARS is critical prior to drug development as it provides detailed insights into the system-specific responses to radiation exposure. In this study, we assessed the effects of 5.8 and 6.5 Gy total-body irradiation (TBI) on multiple organ systems in male and female rhesus macaques through histological evaluations of various tissues, clinical parameters, complete blood counts, and serum biochemistry. A total of 31 nonhuman primates were used to investigate the injury cascade of ARS. Our results demonstrate that TBI induces consistent lung injury along with gastrointestinal and hematopoietic alterations. Of note, severe splenic depletion in non-surviving animals, alongside unexpected tissue-specific responses such as reversed trends in sternum cellularity, were observed. Hematopoietic suppression was dose-dependent, with profound leukopenia, neutropenia, and thrombocytopenia, while red blood cell parameters declined more gradually. Serum biochemistry further revealed dynamic changes in various renal, hepatic, and pancreatic markers. Comparisons between 5.8 and 6.5 Gy exposures, as well as between males and females, revealed differences in injury severity and recovery, underscoring the need to account for both dose and sex in ARS model characterization.

**Supplementary Information:**

The online version contains supplementary material available at 10.1038/s41598-026-49844-x.

## Introduction

The threat of radiological and nuclear incidents continues to cast a shadow over global security, with consequences that extend far beyond the battlefield. Whether resulting from military conflict, terrorist deployment of radiological dispersal devices (RDDs), or accidental nuclear mishaps, exposure to sizable levels of ionizing radiation pose an immediate and profound danger to human health^1–4^. Among the more serious of outcomes is the onset of acute radiation syndrome (ARS), a quickly developing condition caused by radiation injury to critical organ systems following high-dose, partial- or total-body irradiation (PBI or TBI). Its clinical course and severity are largely determined by the absorbed radiation dose and the rate at which it is delivered^5^. At its core, ARS is largely driven by the radiosensitivity of rapidly dividing cells within the bone marrow and gastrointestinal (GI) epithelium, with the inherent sensitivities of neurovascular tissues collectively exacerbating physiological disruption. Consequently, ARS manifests in distinct forms, most commonly the hematopoietic (H-ARS; 2–6 Gy), gastrointestinal (GI-ARS; 6–10 Gy), and neurovascular (NV-ARS; > 10 Gy) subsyndromes^5^.

Accurately assessing radiation exposure and the resulting injuries is challenging due to the body’s complex and dynamic response to ionizing radiation. Radiation damages DNA, induces oxidative stress, and triggers widespread apoptosis and necrosis, yet these effects occur at the cellular and subcellular levels long before overt symptoms appear^6,7^. The prodromal and subsequent latent phases of ARS may span days to weeks, during which molecular and tissue injury accumulates silently. As a result, physical dosimetry offers limited reliability for evaluating injury since the underlying biological damage remains hidden until the acute illness stage develops, which is typically after the prodromal and latent phases^8^. However, despite these pathological hazards, only a handful of regulatory agency-approved medical countermeasures (MCMs) are available.

Fifteen agents are currently approved by the United States Food and Drug Administration (U.S. FDA) for the treatment of acute radiation-induced cytopenias. These include four drugs such as Neulasta, Neupogen, Leukine, and Nplate, and their several biosimilars. Among them, Neulasta and Neupogen and their biosimilars, are approved for the treatment of radiation-induced neutropenia through the stimulation of neutrophil production^9–19^. Similarly, Leukine is approved for the treatment of radiation-induced myelosuppression by promoting the recovery of multiple hematopoietic lineages, including granulocytes, macrophages, and dendritic cells^13,20^. Nplate, on the other hand, is a thrombopoietin receptor agonist approved for the treatment of radiation-induced thrombocytopenia, where it acts to restore platelet counts^21–23^. These MCMs function as radiomitigators for H-ARS, which are administered post-exposure to alleviate hematopoietic injury^24^. In contrast, radioprotectors, which are prophylactic agents administered prior to radiation exposure, remain far less developed. Such agents are of particular importance for the military and civilians alike, where radiation risks in combat or emergency response scenarios demand both immediate and preventative strategies. At present, no FDA-approved radioprotectors are available. Although amifostine is FDA-approved, it is not indicated for use as a radioprotector for ARS; rather, its approved uses are limited to reduce xerostomia (dry mouth) in patients of head and neck cancer receiving radiotherapy and to prevent nephrotoxicity in ovarian cancer patients undergoing cisplatin chemotherapy.

The limited number of FDA-approved MCMs for radiation injury is largely attributable to the logistical challenges of conducting human efficacy trials. To address this, the FDA established the Animal Rule, which allows for approval of MCMs based on well-controlled animal studies when human trials are not feasible or ethical^18,25,26^. Because survival is the primary endpoint in such studies, histopathology serves as a cornerstone of evaluation, providing a direct and scientifically robust means of quantifying the extent of radiation-induced cellular and tissue damage. By defining how organ systems respond to specific radiation doses, histology helps to identify injury thresholds and patterns across ARS subsyndromes, thereby capturing the key pathological features of ARS^27^. Such detailed histopathological evaluations not only illuminate the biological consequences of radiation exposure but also provide a critical foundation for developing more effective and targeted radioprotectors^28,29^.

The rhesus macaque (*Macaca mulatta*) has emerged as the primary large animal model for radiation injury research, providing critical data for the development of MCMs under the FDA Animal Rule^30,31^. With more than 95% genomic homology to humans, rhesus macaques exhibit pathophysiological responses to ionizing radiation that closely parallel those observed in human subjects^32,33^. Their strong molecular, anatomical, and physiological resemblance to humans makes them invaluable for studying clinical and histopathological responses to radiation, enabling in-depth investigations into the development and progression of ARS. Moreover, their relatively long lifespan and comparable requirements for supportive care, such as antimicrobial therapy, nutrition supplementation, and fluid management, in turn strengthen their translational relevance, allowing for comprehensive evaluations of both acute and delayed radiation effects^34,35^. Despite widespread use of this model, sex-specific differences in dose-dependent tissue injury and recovery following ionizing radiation exposure remain relatively uncharacterized. Accordingly, we hypothesized that biological sex may influence the severity and recovery of dose-dependent histopathological injury following near-lethal TBI in rhesus macaques.

In this study, we have further compared the pathological responses of male and female rhesus macaques exposed to two potentially lethal doses of TBI (5.8 and 6.5 Gy). Tissue damage was microscopically evaluated across multiple organs, and physiological responses were assessed through vital signs, complete blood counts (CBCs), and serum biochemistry. TBI was delivered using cobalt-60 (^60^Co) gamma (γ) radiation. Our results show that, as expected, TBI produces widespread systemic injury, most notably within the lymphohematopoietic, GI, and respiratory systems, with the higher 6.5 Gy dose generally causing greater tissue damage. Hematological suppression, evidenced by reductions in platelets and white blood cell (WBC) subsets, underscores the extensive physiological stress induced by radiation exposure. Minimal effects were observed in vital signs and serum biochemistry parameters. Although many responses were dose-dependent, certain tissues and CBC endpoints exhibited unexpected patterns, and recovery dynamics varied by sex in both severity and timing. Collectively, these findings emphasize the dose-, sex-, and organ-specific complexities underlying the pathophysiology of ARS.

## Materials and methods

### Experimental design

This study included male and female rhesus macaques to evaluate, principally via a histopathologic approach, the effects of two potentially lethal doses of ionizing radiation exposure. The objective was to characterize radiation-induced injury across multiple organ systems and better understand how the body responds to acute, high-dose exposure in both sexes^36–38^. Animals were exposed to either 5.8 or 6.5 Gy total-body ^60^Co γ-radiation. These doses correspond to the LD_30/60_ and LD_50/60_ thresholds for rhesus macaques, respectively^39,40^. The 5.8 Gy cohort included 15 animals (10 males, 5 females) and the 6.5 Gy cohort included 16 animals (6 males, 10 females). Survival outcomes and hematopoietic recovery were assessed for 60 days post-irradiation using CBC analyses and additional physiological measures. A select number of animals became moribund and were euthanized prior to reaching the 60-day endpoint^39,40^. No animals experienced mortality prior to euthanasia. Necropsy was performed on all animals, and tissues from various organ sites were systematically collected for histological analysis.

### Animals

A total of 31 naïve rhesus macaques (*Macaca mulatta*, Chinese sub-strain) were obtained from Primate Products, Inc. (Miami, FL, USA) and underwent a minimum of a seven-week quarantine period prior to study initiation. Animal quarantine details have been provided in an earlier study^39^. The final study group consisted of healthy male and female animals ranging in age from 3.6 to 6.3 years and weighing between 4.05 and 8.95 kg. Animals were acquired at staggered time points to meet the age and weight criteria outlined in the Institutional Animal Care and Use Committee (IACUC)-approved protocol and were therefore not randomized across groups. All animals were stratified by sex and weight gain observed during the quarantine period, after which they were then assigned to their respective study groups.

Animals were housed individually in an Association for Assessment and Accreditation of Laboratory Animal Care (AAALAC) International-approved vivarium. Housing conditions, environmental enrichment, and overall care during the study period were conducted in accordance with institutional protocols and have been previously described^41^. All animal procedures were approved by the IACUC of the Armed Forces Radiobiology Research Institute (AFRRI), Uniformed Services University of the Health Sciences (USUHS), and the Department of War Animal Care and Use Review Office (ACURO). The study was carried out in full compliance with the *Guide for the Care and Use of Laboratory Animals*^42^ and ARRIVE (Animal Research: Reporting of In Vivo Experiments) guidelines.

### Irradiation

A maximum of eight animals were irradiated per day according to the experimental schedule. To minimize the risk of radiation-induced emesis, food was withheld for approximately 12–18 h prior to irradiation, while water remained available ad libitum. Sedation was administered 30–40 min before radiation exposure through an intramuscular (*im*) injection of ketamine hydrochloride (Zoetis Inc., Kalamazoo, MI, USA) at a dose of 10–15 mg/kg. As body weight and size influence the absorbed dose and subsequent injury severity, all animals in this study were randomized by weight. Prior to irradiation, the abdominal widths of each animal were measured using digital calipers (iGaging, San Clemente, CA, USA). These measurements were provided to the attending dosimetrist and were used to accurately deliver the precise dose of radiation to the midline of the animal. Based on these measurements, the animals were paired with a similar-sized (within 1 cm abdominal measurement) partner and were irradiated together. If there was no suitable animal to make a pair, the animal was irradiated alone, and a phantom was used in place of another animal. Each animal was positioned upright in a separate Plexiglas box, oriented in opposite directions to ensure uniform exposure, and secured by tying the limbs to an external cleat. This strategy was used to ensure that each animal received the same approximate radiation dose regardless of body weight.

Once properly positioned, the boxes were transported by cart via elevator to the irradiation facility. If needed, a 0.1–0.3 mL booster dose of *im* ketamine (100 mg/mL) was administered to limit movement during exposure. Animals received 5.8 or 6.5 Gy total-body midline ^60^Co γ-radiation, delivered bilaterally at a targeted rate of 0.6 Gy/min, with actual rates between 0.58 and 0.59 Gy/min, respectively, directed at both sides of the abdominal core. The full dosimetry methods of the irradiation procedure have been outlined in previous studies^41,43^. The radiation field at the site of animal placement was consistent, with variation not exceeding ± 1.5%. Dosimetry measurements for phantoms and animals alike were primarily obtained using the alanine/electron paramagnetic resonance (EPR) technique as described earlier^44^. During the irradiation procedure, the nonhuman primates (NHPs) were continuously monitored through closed-circuit video surveillance. Following exposure, all NHPs were returned to the vivarium and placed back in their designated home cages, where they were closely observed until the sedative effects subsided and normal mobility was restored.

### Blood sample collection

Blood samples were performed on fully conscious animals positioned in restraint chairs utilizing a pole-and-collar system to minimize movement and mitigate handling-related stress. To ensure animals were comfortable with the procedure, they were acclimated to the restraint apparatus over several weeks prior to study initiation. Positive conditioning methods were employed throughout training, with animals receiving treats before, during, and after each session, to encourage cooperation and reward behavior. To collect blood, peripheral venipuncture was performed. The chosen collection site was either the saphenous vein in the lower leg or the cephalic vein in the lower arm, approximately 1–3 h after the animals were fed. Prior to venipuncture, a tourniquet was applied, and the site was disinfected using a 70% isopropyl alcohol wipe to maintain aseptic conditions and improve vein visibility. A 3 mL disposable luer-lock syringe with a 25-gauge needle was used to collect the desired amount of blood^37^. Following sample collection, light pressure with sterile gauze was applied to the venipuncture site to facilitate hemostasis and minimize hematoma formation.

Blood samples for CBC analysis were collected in EDTA tubes (Sarstedt Inc., Newton, NC, USA) on pre-irradiation days -7 and -3, followed by post-irradiation collections on days 1, 2, and every other day from day 4 through day 30. Additional post-irradiation samples were collected on days 34, 38, 42, 50, and 60. For biochemistry analysis, collected blood samples were processed as described earlier^37^. The resulting serum was aliquoted into specimen tubes until analysis. Biochemistry assessments were conducted on day -3 (pre-irradiation) and subsequently on days 2, 28, 38, 50, and 60 post-irradiation.

### CBC and serum biochemistry analysis

CBC analysis was performed using a Bayer Advia-120 Hematology Analyzer (Siemens Medical Solutions, Malvern, PA, USA), while serum biochemistry analysis was performed using a Vitros 350 Automatic Biochemistry Analyzer (Ortho Clinical Diagnostics, Markham, ON, Canada)^37,45^. The following 11 blood parameters were analyzed: WBCs, red blood cells (RBCs), hematocrit (HCT), hemoglobin (HGB), platelets, and absolute counts for neutrophils, lymphocytes, monocytes, eosinophils, basophils, and reticulocytes.

Additionally, the following 23 serum biochemistry parameters were analyzed: sodium, potassium, chloride, carbon dioxide (CO_2_), glucose, blood urea nitrogen (BUN), creatinine, calcium, phosphorus, total protein, albumin, total bilirubin, alkaline phosphatase (ALKP), lactate dehydrogenase (LDH), aspartate aminotransferase (AST), alanine aminotransferase (ALT), gamma-glutamyl transferase (GGT), amylase, lipase, cholesterol, direct high-density lipoprotein cholesterol (HDLC), triglycerides, and uric acid.

### Supportive care

Throughout the post-procedural period, all animals were observed at least twice daily (BID) to monitor for any signs of health complications. Supportive care decisions were informed by CBC results and routine behavioral and physical assessments at the cage side^37,46^. A detailed description of supportive care can be found in an earlier publication^36^. Other supportive care measures included rehydration fluid therapy, alternative antibiotics, antipyretics, antidiarrheal agents, analgesics, antiemetics, mucosal ulcer treatment, and nutritional enrichment^36,39^. No blood products were administered during the study.

### Clinical evaluation

All animals were monitored prior to irradiation and for a period of 60 days afterward, with survival designated as the primary outcome measure. Observations for indicators of pain or distress were conducted at least BID by either husbandry personnel or research staff. From days 10–20 following irradiation, monitoring frequency increased to three times daily (TID), spaced roughly 8–10 h apart. Accounts of the observation procedures and standard protocols have been published previously^47^.

### Euthanasia

Euthanasia was performed in accordance with the *Guide for the Care and Use of Laboratory Animals*, the approved IACUC protocol, and the American Veterinary Medical Association’s (AVMA) Guidelines for Animal Euthanasia^42,48^. Animals were euthanized upon reaching moribund status, defined as a condition of no recovery. Moribundity served as a proxy for mortality, and euthanasia was carried out to alleviate any undue suffering or distress^49,50^. Criteria for moribundity can be found in a previously published study^41^. Additionally, a veterinarian assessed dysfunction across major organ systems, including respiratory, GI, urogenital, nervous, and integumentary systems to inform the moribundity determination^36^. No euthanasia decision was made solely based on symptom clusters without veterinarian consultation. Decisions regarding moribundity and subsequent euthanasia involved collaborative input from veterinarians, veterinary technicians, the principal investigator, research study personnel, and animal care staff. In brief, moribund animals were first sedated with ketamine hydrochloride (5–15 mg/kg, *im*), maintained with isoflurane (Baxter Healthcare Corporation, Deerfield, IL, USA) (1–5%) with oxygen at 1–4 L per minute via mask. Then euthanasia was performed with pentobarbital sodium (Virbac AH Inc., Fort Worth, TX, USA) administered intravenously or intra-cardiac. The detailed euthanasia protocol can be found earlier^41^.

### Necropsy and histopathology

Necropsy examinations were conducted on animals that were euthanized upon reaching moribund status during the study, as well as on those euthanized at the designated study endpoint. Tissue collection was performed under the supervision of a veterinary pathologist, with all visible gross abnormalities documented during the procedure. Eleven tissues were collected from each animal, including the sternum, spleen, duodenum, jejunum, ileum, large intestine, liver, kidney, urinary bladder, lung, and heart. All tissue samples were placed in histology cassettes, transferred to appropriate containers, and preserved in 10% buffered zinc formalin. Once fixed, tissues were processed for paraffin embedding, sectioned, and stained with hematoxylin and eosin (H&E) for slide preparation by Histoserv, Inc. (Germantown, MD, USA).

A veterinary pathologist performed histopathological scoring and morphometric evaluations. Tissue pathology was graded using a semi-quantitative scale ranging from 0 to 5, with 0 indicating no abnormality and 5 representing severe damage. The scale was defined as follows: 0 = none; 1 = minimal (barely perceptible); 2 = mild; 3 = moderate (up to 50% of the tissue affected); 4 = marked (51–90% affected); and 5 = severe (> 90% affected). This scoring system represents the standard approach used in veterinary toxicologic pathology and ensures consistency and comparability across histopathological evaluations with most, if not all, pathologist grading. Grading was based on the pathologist’s interpretation of the extent and severity of visible microscopic changes observed in the stained tissue sections. High-resolution digital images of H&E-stained sections were acquired using a Zeiss Axioscan slide scanner and analyzed with Zeiss Zen software (Carl Zeiss Meditech, Inc., Dublin, CA, USA). Additional microscopic examination was carried out using an Olympus IX73 microscope (Olympus, Center Valley, PA, USA) using cellSens image acquisition software. All histological and morphometric assessments were accomplished by a single pathologist and conducted under blind conditions to prevent bias related to treatment group identity. In the images, star landmarks were used to indicate histological features surrounding the denoted area of interest and arrows pointing to specific localized histological features.

### Data analysis

Vital signs, CBC, serum biochemistry, and histopathology data were evaluated for radiation-induced changes over time. One-way analysis of variance (ANOVA) tests were used for exploratory comparisons to assess differences in each radiation dose group at each time point. A minimum of three animals per group was required at each time point to ensure valid statistical analysis. If either group had fewer than three animals for a given comparison, statistical analysis was not performed. All error bars represent standard deviations.

CBC data was further assessed to account for repeated measures within animals with linear mixed-effect models applied for each parameter. Fixed effects for each model included radiation dose (5.8 vs. 6.5 Gy), time, and their interaction. Animal IDs were included as a random intercept. Estimated marginal means were then calculated from each mixed-effects model to obtain model-adjusted means for radiation dose at each time point. Pairwise contrasts were then performed to compare 5.8 Gy vs. 6.5 Gy at each time point. *P*-values were adjusted using the Benjamini Hochberg false discovery rate (FDR) method. Lastly, histopathology data were scored on a 6-point Likert scale, as previously discussed, with 0 indicating normal tissue and 5 indicating severe changes. Mann–Whitney U tests were performed to assess significant differences in tissue lesions between radiation dose groups, non-surviving animals in the 5.8 Gy group compared to non-surviving animals in 6.5 Gy, and 5.8 Gy surviving animals compared to 6.5 Gy surviving animals. The one-way ANOVA and Mann–Whitney U tests were performed using SPSS v.28 (IBM Corp., Armonk, NY, USA), while the linear mixed-effects models were performed in R version 4.4.1. *P*-values of less than 0.05 were considered statistically significant.

## Results

The study assessed organ-specific pathological responses in male and female rhesus macaques exposed to two potentially lethal TBI doses (5.8 and 6.5 Gy) using primarily microscopic and clinical procedures. Supplementary Fig. 1 presents Kaplan–Meier survival curves for animals exposed to 5.8 and 6.5 Gy, with additional stratification by sex, to allow visualization of group-specific responses to TBI. No significant differences in survival were observed among various groups (male vs. female and 5.8 vs. 6.5 Gy), likely due to the small sample sizes and the relatively similar radiation doses. Survival at study completion was 66.67% for males and 60% for females, with identical rates observed in both the 5.8 and 6.5 Gy groups. To supplement the histopathology analysis, key vital signs, CBCs, and serum biochemistry parameters were evaluated to better characterize the progression of radiation injury and its relationship to organ-specific pathology. Using both sexes allowed us to capture holistic differences in physiological responses based on the two TBI doses, ensuring a more comprehensive evaluation of radiation injury across the study cohort.

### Histopathology

**Fig. 1:**
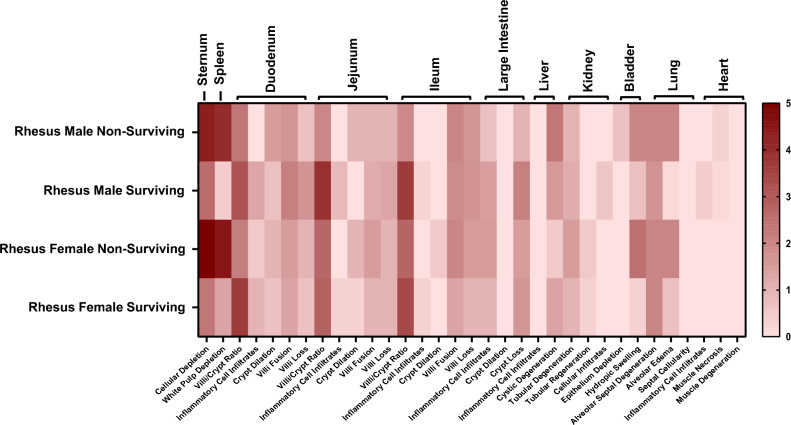
Heatmap of average histopathology scores of various organs collected from male and female rhesus macaques exposed to 5.8 Gy TBI. Data for each section is presented as the mean for each group. Image was generated using GraphPad Prism version 10.5.0 statistical software.

**Fig. 2:**
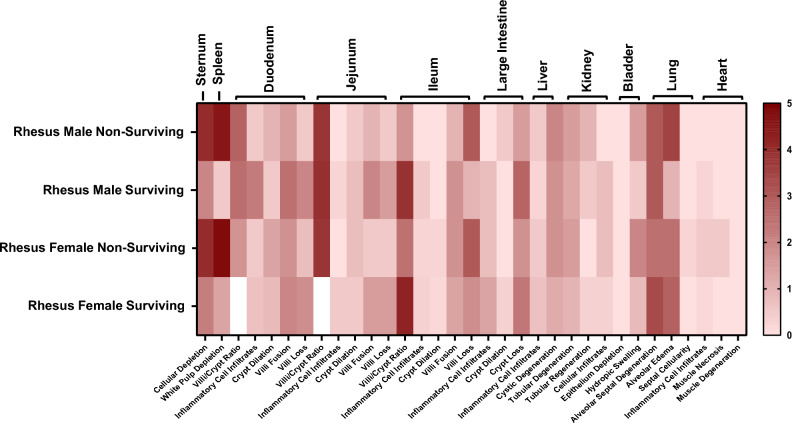
Heatmap of average histopathology scores of various organs collected from male and female rhesus macaques exposed to 6.5 Gy TBI. Data for each section is presented as the mean for each group. Image was generated using GraphPad Prism version 10.5.0 statistical software.

Overall pathological findings from each animal at necropsy were documented and summarized in Supplementary Tables 1–6. Tissues of all irradiated animals, “male non-surviving,” “male surviving,” “female non-surviving,” and “female surviving,” were examined and reported here. Tissues collected from surviving and non-surviving NHPs of both radiation doses, as well as healthy animal tissues, were compared. The pathological findings in surviving animals sacrificed on day 60 likely reflect a combination of persistent radiation injury and subsequent recovery processes. A heatmap of mean histopathology scores across organs for the 5.8 Gy group is shown in Fig. 1, with each lesion represented by its average score; the corresponding heatmap for the 6.5 Gy group is shown in Fig. 2. As described previously, a veterinary pathologist performed histopathological scoring under blinded conditions using a semi-quantitative scale from 0 (no abnormality) to 5 (severe), where 1 = minimal, 2 = mild, 3 = moderate, and 4 = marked. Mann–Whitney U tests were conducted to evaluate significant differences in histopathological scores both across radiation dose groups (5.8 vs. 6.5 Gy) and between surviving and non-surviving animals within each dose group (Supplementary Table 7). In addition, select tissues from unirradiated NHPs were processed and examined as controls, providing a baseline for comparison with the acutely irradiated animals. Representative micrographs of these control tissues, labeled as “healthy” within the panels, reflect their normal state at the time of necropsy. Furthermore, magnification levels differ according to the region of interest. Key findings from selected tissues of interest in surviving and non-surviving subjects are presented in Tables 1 and 2, respectively. Specifically, 6.5 Gy TBI consistently caused more severe damage than 5.8 Gy TBI, with this dose-dependent effect being more pronounced in the lungs, spleen, and certain sections of the small intestine, such as the jejunum and ileum. However, some organs, such as the sternum, showed the reversed trend, indicating that the effects of radiation exposure varied unpredictably across different tissue types.

**Table 1:** Key histopathological findings comparing surviving subjects exposed to 5.8 and 6.5 Gy, stratified by sex.

	Surviving males		Surviving females	
	5.8 Gy	6.5 Gy	5.8 Gy	6.5 Gy
Jejunum	None-to-minimal inflammatory cellular infiltration	None-to-minimal inflammatory cellular infiltration; Increased villi fusion and crypt dilation	None-to-minimal inflammatory cellular infiltration; Minimal villi loss	Severe villi-to-crypt ratio; Minimal-to-mild villi loss; Minimal-to-mild villi fusion and crypt dilation; None-to-minimal inflammatory cellular infiltration
Ileum	Minimal-to-mild villi loss*	Minimal villi loss	Moderate-to-marked villi-to-crypt ratio	Marked-to-severe villi-to-crypt ratio; Greater villi loss
Duodenum	Minimal-to-mild villi-to-crypt ratio and villi loss	Mild villi-to-crypt ratio and villi loss	None-to-minimal villi-to-crypt ratio and villi loss; Minimal inflammatory cellular infiltration*	Mild villi-to-crypt ratio and villi loss; None-to-minimal inflammatory cellular infiltration
Large Intestine	Minimal-to-mild inflammatory cellular infiltration*; Mild-to-moderate crypt loss	None-to-minimal inflammatory cellular infiltration; Mild-to-moderate crypt loss	Minimal inflammatory cellular infiltration; Minimal-to-mild crypt loss	Minimal-to-mild inflammatory cellular infiltration; None-to-minimal crypt dilation; Mild-to-moderate crypt loss
Spleen	None-to-minimal depletion	None-to-minimal depletion	Minimal-to-mild white pulp cellular depletion	Minimal-to-mild white pulp cellular depletion
Lungs	Minimal-to-mild alveolar septal degeneration; None-to-minimal alveolar edema	Mild-to-marked alveolar septal degeneration; Minimal alveolar edema	Mild alveolar septal degeneration; None-to-minimal alveolar edema	Mild-to-moderate alveolar septal degeneration; Mild-to-moderate alveolar edema
Sternum	Mild-to-moderate cellular depletion*	Mild cellular depletion	Mild-to-moderate cellular depletion*	Mild-to-moderate cellular depletion

Organs including the liver, kidneys, urinary bladder, and heart exhibited unclear dose-dependent trends and were therefore excluded from this analysis. An asterisk (*) denotes unexpected findings.

**Table 2:** Key histopathological findings comparing non-surviving subjects exposed to 5.8 and 6.5 Gy, stratified by sex.

	Non-surviving males		Non-surviving females	
	5.8 Gy	6.5 Gy	5.8 Gy	6.5 Gy
Jejunum	Minimal villi loss*	None-to-minimal villi loss; High villi-to-crypt ratio	Minimal villi loss*	None-to-minimal villi loss; High villi-to-crypt ratio
Ileum	Minimal-to-mild villi loss; Mild villi fusion*	Moderate villi loss; Minimal villi fusion	Minimal-to-mild villi loss; Mild villi fusion*	Moderate villi loss; None-to-minimal villi fusion
Duodenum	No inflammatory cellular infiltration	None-to-minimal inflammatory cellular infiltration	Minimal villi loss	Minimal-to-mild villi loss
Large Intestine	None-to-minimal inflammatory cellular infiltration*	No inflammatory cellular infiltration; None-to-minimal crypt dilation	Minimal-to-mild inflammatory cellular infiltration*; Minimal-to-mild crypt loss	Minimal-to-mild crypt loss
Spleen	Marked white pulp cellular depletion	Marked-to-severe white pulp cellular depletion	Marked-to-severe white pulp cellular depletion	Marked-to-severe white pulp cellular depletion
Lungs	Mild alveolar septal degeneration; Mild alveolar edema	Moderate alveolar septal degeneration; Moderate alveolar edema	Mild alveolar septal degeneration; Mild alveolar edema	Mild-to-moderate alveolar septal degeneration; Mild-to-moderate alveolar edema
Sternum	Marked-to-severe cellular depletion*	Marked cellular depletion	Severe cellular depletion*	Marked cellular depletion

Organs including the liver, kidneys, urinary bladder, and heart exhibited unclear dose-dependent trends and were therefore excluded from this analysis. An asterisk (*) denotes unexpected findings.

#### Lymphohematopoietic system/sternum

**Fig. 3:**
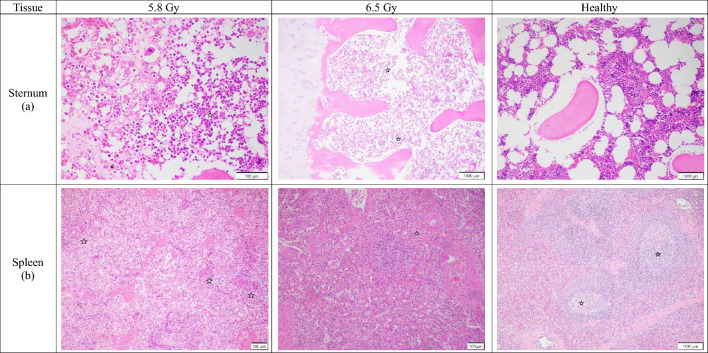
Comparison of sternum and spleen tissue sections collected from male and female rhesus macaques exposed to 5.8 or 6.5 Gy, as well as healthy tissue. (**a**) 5.8 Gy (NHP ID RA1272M, scale bar = 100 μm) There is marked cellular depletion with minimal nucleated cells of various types; 6.5 Gy (NHP ID RA2693M, scale bar = 1000 μm) There is marked cellular loss throughout the marrow space with minimal cellular presence (stars); Healthy (NHP ID 14041532F, scale bar = 1000 μm) There are no abnormalities noted. (**b**) 5.8 Gy (NHP ID RA1671F, scale bar = 100 μm) There is severe depletion of white cellular components throughout the spleen parenchyma (stars); 6.5 Gy (NHP ID RA2688M, scale bar = 100 μm) There is severe cellular depletion of the white pulp (star); Healthy (NHP ID 14041532F, scale bar = 1000 μm) The spleen is characterized by well-formed white pulp (stars), indicative of normal lymphoid tissue.

Sternum cellular depletion showed a consistent, but unexpected, trend in all groups (Fig. 3a). The lower dose of 5.8 Gy resulted in more severe cellular depletion than the higher dose of 6.5 Gy. In non-surviving subjects, the mean depletion for the 5.8 Gy group was consistently higher than for the 6.5 Gy group (males: mean grade 4.5 vs. 4; females: mean grade 5 vs. 4). The same reversed trend was seen in surviving subjects, though the differences were minimal (males: mean grade 2.6 vs. 2; females: mean grade 2.7 vs. 2.2).

#### Lymphohematopoietic system/spleen

White pulp cellular depletion in the spleen was severe in all non-surviving subjects, with only a slight, dose-dependent increase observed at 6.5 Gy (Fig. 3b). Non-surviving males at the higher dose showed marked to severe depletion (mean grade 4.5) compared to marked depletion at the lower dose (mean grade 4). The same minimal increase was seen in non-surviving females. In contrast, there was no observable difference in white pulp depletion between 5.8 and 6.5 Gy surviving subjects, with both showing very low levels of depletion (males: mean grade 0.43 vs. 0.5; females: mean grade 1.3 vs. 1.3).

#### Gastrointestinal system/small intestine/duodenum

**Fig. 4:**
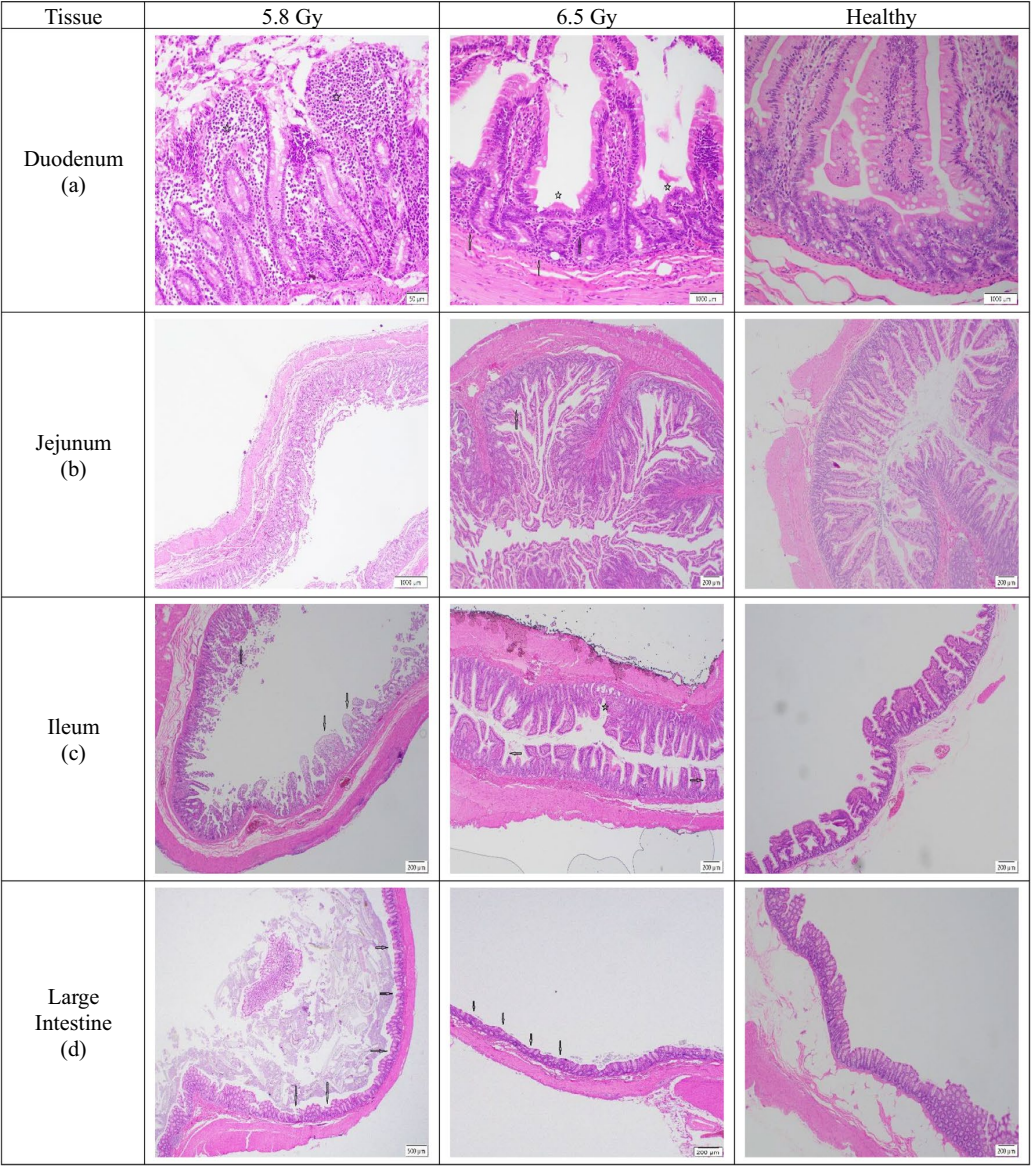
Comparison of duodenum, jejunum, ileum, and large intestine tissue sections collected from male and female rhesus macaques exposed to either 5.8 or 6.5 Gy, as well as healthy tissue. (**a**) 5.8 Gy (NHP ID RQ9719M, scale bar = 50 μm) There is moderate inflammatory cellular infiltrates expanding moderately fused villi (stars); 6.5 Gy (NHP ID RA1463F, scale bar = 1000 μm) There is moderate loss of villi with empty space between remaining villi (stars). There is also minimal inflammatory cell infiltrates composed of polymorphonuclear cells (arrows); Healthy (scale bar = 1000 μm) The tissue is essentially normal. (**b**) 5.8 Gy (NHP ID RA1352M, scale bar = 1000 μm) The tissue is essentially normal with minimal crypt dilation, villi loss, and villi fusion; 6.5 Gy (NHP ID RA2688M, scale bar = 200 μm) There is minimal villi fusion (arrows), otherwise the tissue is essentially normal with minimal autolysis; Healthy (scale bar = 200 μm) Tissue appears essentially normally. (**c**) 5.8 Gy (NHP ID RA1272M, scale bar = 200 μm) There is mild villi fusion (arrows) and the tissue is overall mildly autolyzed; 6.5 Gy (NHP ID RA2693M, scale bar = 200 μm) There is minimal villi fusion (arrows) and minimal crypt dilation (star); Healthy (scale bar = 200 μm) There are areas of minimal to mild crypt fusion. (**d**) 5.8 Gy (NHP ID RA2247F, scale bar = 500 μm) There is regionally moderate crypt loss (arrows); 6.5 Gy (NHP ID RA0443F, scale bar = 200 μm) There is marked crypt loss (arrows); Healthy (scale bar = 200 μm) The crypt gland height is minimally uneven.

Subjects exposed to 6.5 Gy generally showed more damage than those from the 5.8 Gy group, though some inconsistent trends were noted (Fig. 4a). In surviving subjects, the higher-dose group showed a slightly greater villi-to-crypt ratio, which was consistent with increased villi loss (males: mean grade 2 vs. 1.7; females: mean grade 2 vs. 0.67). A similar trend of greater villi loss at the higher dose was observed in non-surviving females (mean grade 1.8 vs. 1). However, villi loss was similar in non-surviving males between both groups, and there were no differences in crypt dilation across any subsets. Inflammatory cellular infiltration was also irregular; non-surviving males at 6.5 Gy showed a minimal increase in infiltrates (mean grade 0.5) compared to the 5.8 Gy group (mean grade 0), but no difference was seen in non-surviving females. In contrast, the expected trend was reversed in surviving females, where the lower-dose group showed slightly more inflammation (mean grade 1) than the higher-dose group (mean grade 0.67).

#### Gastrointestinal system/small intestine/jejunum

6.5 Gy subjects showed more damage than 5.8 Gy subjects, though some findings were unexpectedly reversed, especially in non-surviving subjects. The villi-to-crypt ratio was consistently higher in the 6.5 Gy group for all subsets except for surviving males. The findings for villi loss were inconsistent; non-surviving subjects from the 6.5 Gy group showed less villi loss (mean grade 0.5) than those at 5.8 Gy (mean grade 1), which is opposite of what would be expected with higher radiation doses. This may be because non-surviving animals were euthanized upon reaching moribund status, before the full extent of tissue damage could be seen. However, the higher-dose surviving female group showed greater villi loss (mean grade 1.5) than the lower-dose group (mean grade 1). Villi fusion and crypt dilation followed inconsistent patterns in non-surviving subjects but showed dose-dependent increases among surviving subjects (Fig. 4b). Inflammatory cellular infiltration was absent in all non-surviving animals. In surviving subjects, however, the trend was irregular, with the 6.5 Gy males showing less inflammation (mean grade 0.25) than 5.8 Gy males (mean grade 0.86), while the opposite was true for females (mean grade 0.5 vs. 0.33).

#### Gastrointestinal system/small intestine/ileum

Subjects exposed to 6.5 Gy generally showed more severe damage than those exposed to 5.8 Gy. The villi-to-crypt ratio was slightly higher in the 6.5 Gy surviving females (mean grade 4.2) than in the 5.8 Gy surviving females (mean grade 3.4), but no difference was seen in the other groups. Villi loss was significantly higher in non-surviving subjects at the higher dose (mean grade 3) compared to the lower dose (males: mean grade 1.7; females: mean grade 1.5) (*p* = 0.030, effect size = −0.698). However, surviving males showed less villi loss at the higher dose (mean grade 1) than at the lower dose (mean grade 1.7), while surviving females showed the opposite trend. Villi fusion was unexpectedly lower in both non-surviving male (mean grade 1) and female (mean grade 0.88) subjects at 6.5 Gy than in their 5.8 Gy counterparts (male and female mean grades 2), possibly because the animals were moribund and euthanized before the full damage could be seen (Fig. 4c). There were no differences in villi fusion in any of the surviving groups. Similarly, there were no discernible differences in crypt dilation or inflammatory cellular infiltration between any of the groups.

#### Gastrointestinal system/large intestine

Inflammatory cellular infiltration was unexpectedly lower in the 6.5 Gy non-surviving subjects compared to those at 5.8 Gy, possibly because they were moribund and euthanized before inflammation could fully develop. In non-surviving males, the mean score was 0 at 6.5 Gy compared to 0.67 at 5.8 Gy. This trend was reversed in surviving subjects; 6.5 Gy females had slightly more inflammation (mean grade 1.33) than 5.8 Gy females (mean grade 1), while 6.5 Gy males had less inflammation (mean grade 0.75) than their 5.8 Gy counterparts (mean grade 1.4). Crypt dilation was minimal or absent in all groups, with slightly more seen at the higher dose in non-surviving males (mean grade 0.5) and surviving females (mean grade 0.33). For crypt loss, the 6.5 Gy group showed a slightly greater loss in surviving subjects (males: mean grade 2.8; females: mean grade 2.3) compared to the 5.8 Gy group (males: mean grade 2.1; females: mean grade 1.7), which is consistent with the dose increase (Fig. 4d). However, the difference was very small in non-surviving females (mean grade 1.8 vs. 1.5). Non-surviving male crypt loss could not be analyzed due to tissue degradation.

#### Digestive system/liver

**Fig. 5:**
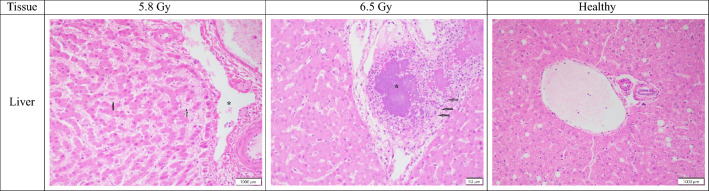
Comparison of liver tissue sections collected from male and female rhesus macaques exposed to 5.8 or 6.5 Gy, as well as healthy tissue. 5.8 Gy (NHP ID RA1413M, scale bar = 1000 μm) There is mild cystic dilation centered on triportal areas (star). Sinusoidal spaces are mildly expanded by eosinophilic proteinaceous material (arrows); 6.5 Gy (NHP ID RA2693M, scale bar = 50 μm) There is foci of minimal inflammatory cell infiltrates composed of monocytes and polymorphonuclear cells (stars). There is mild cystic dilation centered on triportal areas (stars); Healthy (scale bar = 1000 μm) The liver tissue section is essentially normal.

Subjects exposed to 6.5 Gy showed minimal histopathologic increases in hepatic inflammatory cellular infiltrates compared with those exposed to 5.8 Gy. Although the overall differences between doses were subtle, a slight dose-dependent increase was consistently observed. This trend was seen in both non-surviving and surviving subjects. While most liver sections showed no clear association between TBI dose and cystic degeneration, non-surviving females at 6.5 Gy showed a minimal increase in these changes (grades 1–3, mean 1.8) compared to the changes (grades 0–1, mean 0.5) seen in the 5.8 Gy group, though the significance is uncertain. These cystic changes were characterized by dilated spaces likely due to previous endothelial cell damage (Fig. 5). Additionally, two male subjects from the non-surviving 6.5 Gy cohort (RA2688M and RA2693M) also showed bacterial colonies (both minimal, grades 1), suggesting the TBI-induced damage at this higher dose may have made the liver more susceptible to a bacterial infection.

#### Urinary system/kidney

**Fig. 6:**
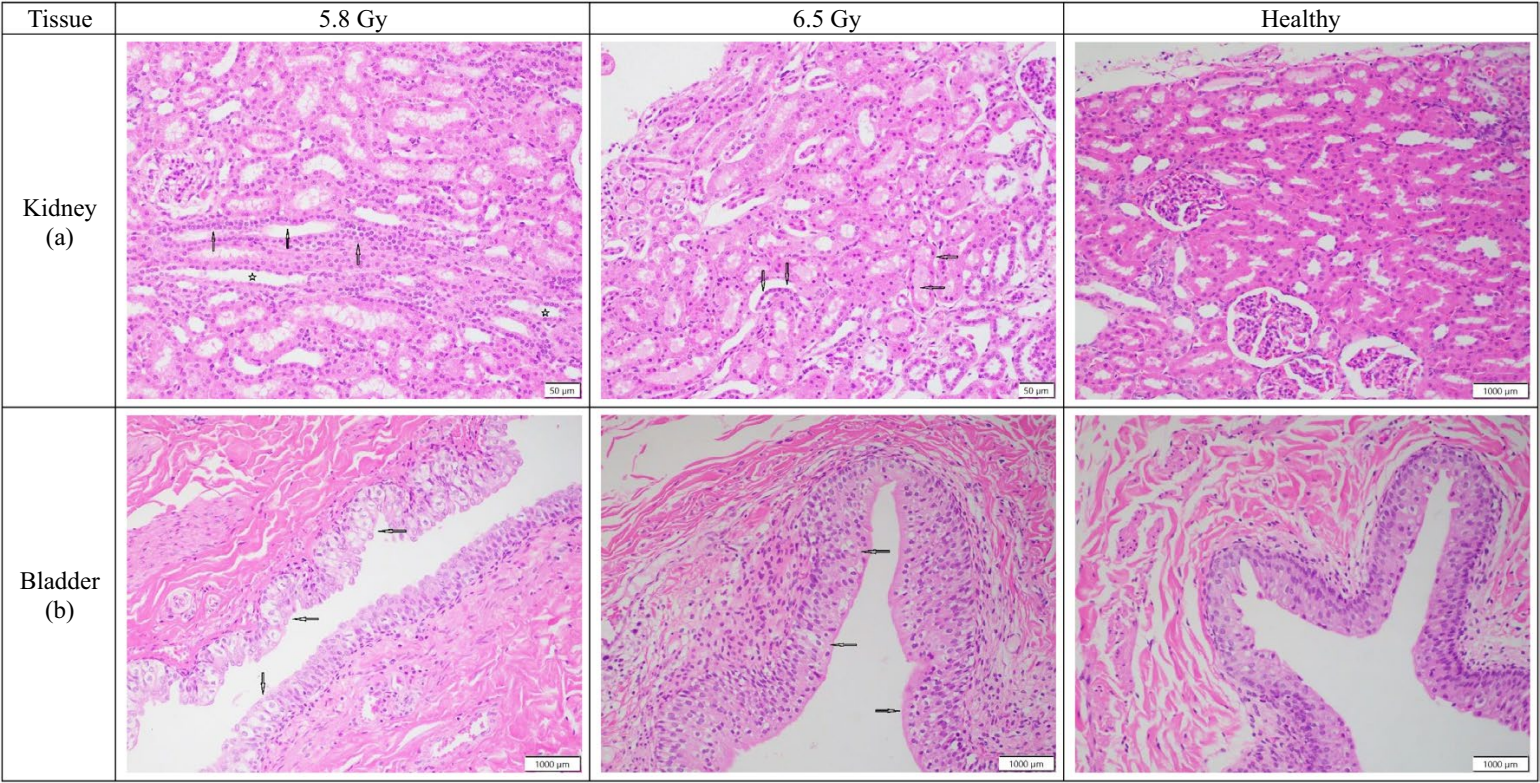
Comparison of kidney and bladder tissue sections collected from male and female rhesus macaques exposed to 5.8 or 6.5 Gy, as well as healthy tissue. (**a**) 5.8 Gy (NHP ID RA0762F, scale bar = 50 μm) There are areas of minimal tubular regeneration with larger crowded epithelium (arrows), and areas of degeneration with attenuated epithelium and dilated tubules (stars); 6.5 Gy (NHP ID RA2688M, scale bar = 50 μm) There are areas of mild tubular degeneration with attenuated epithelium with shrunken dark nuclei and enlarged tubular lumen (arrows); Healthy (scale bar = 1000 μm) There is overall mild dilation of tubular lumen. (**b**) 5.8 Gy (NHP ID RA1272M, scale bar = 1000 μm) Urothelium is mildly depleted with only one to two cell layers thick in some areas (arrows); 6.5 Gy (NHP ID RA2693M, scale bar = 1000 μm) There is mild intracellular swelling of urothelium with clear areas surrounding nuclei in some areas (arrows); Healthy (scale bar = 1000 μm) Urothelium is uniformly three to four cell layers thick with minimal histological changes.

Across all subjects and groups, the kidneys showed minimal to mild tubular degeneration, characterized by dilated tubules with flattened epithelial cells (Fig. 6a). While this damage was widespread, there were only slight, dose-dependent differences. The 6.5 Gy non-surviving males showed a slightly higher score of tubular degeneration (mean grade 1.5) than the 5.8 Gy group (mean grade 1), which was a trend not seen in the other groups. Similarly, tubular regeneration was minimal and only varied slightly by group. In contrast to males, non-surviving females showed greater tubular regeneration at the lower dose, while surviving females showed no difference between groups. Inflammatory infiltration was generally absent, with the only notable exception being a minimal increase in 6.5 Gy non-surviving females (mean grade 0.75) compared to the 5.8 Gy group (mean grade 0). Separately, a single non-surviving male in the 5.8 Gy group (RA1413M) exhibited multifocal bacterial colonies composed of cocci (minimal, grade 1), an isolated finding without evidence of broader group involvement.

#### Urinary system/bladder

Histological analysis of the urinary bladder showed very little difference between the 5.8 and 6.5 Gy groups (Fig. 6b). There was no discernible trend in urothelial depletion, as almost all samples showed no depletion (mean grade 0). The only exception was one subject in the 5.8 Gy non-surviving male cohort, which had mild depletion (grade 2). This isolated finding could not be explained by pathology alone as it needs to be evaluated using other clinical assessments. The trend in urothelial hydropic degeneration was inconsistent and not clearly related to the TBI dose. In non-surviving males, the 5.8 Gy group had slightly more degeneration (mean grade 2) than the 6.5 Gy group (mean grade 1.5). However, non-surviving females at both doses showed the exact same average degeneration score (mean grade 2). In the surviving subjects, degeneration was minimal overall, with both male groups showing similar levels of change (mean grade 0.71 vs. 0.5). A slight increase was observed in 6.5 Gy surviving females (mean grade 0.83) compared to those at 5.8 Gy (mean grade 0.33), but the cause of this irregularity is uncertain.

#### Respiratory system/pulmonary tissue/lung

**Fig. 7:**
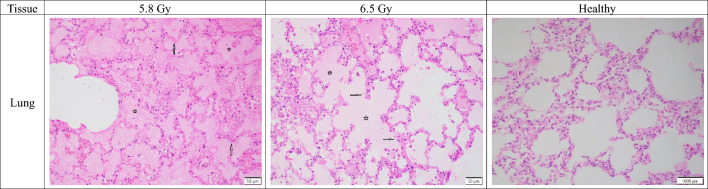
Comparison of lung tissue sections collected from male and female rhesus macaques exposed to 5.8 or 6.5 Gy, as well as healthy tissue. 5.8 Gy (NHP ID RA1272M, scale bar = 50 μm) Alveolar septa is moderately degenerated (arrows) and there is moderate alveolar edema (stars); 6.5 Gy (NHP ID RA1493F, scale bar = 50 μm) Alveolar septum is moderately degenerated (arrows) and there is marked edema in the alveoli (stars); Healthy (NHP ID 14041532F, scale bar = 1000 μm) There are no abnormalities noted.

Lung sections from subjects receiving 6.5 Gy consistently showed more severe damage than those from subjects receiving 5.8 Gy. This dose-dependent relationship was observed in all subject groups, regardless of sex or survival outcome. Both alveolar septal degeneration and alveolar edema were more pronounced in the 6.5 Gy group (Fig. 7). For example, non-surviving males at 6.5 Gy showed moderate septal degeneration (mean grade 3) compared to the mild changes in the 5.8 Gy group (mean grade 2). Similarly, surviving males at 6.5 Gy showed septal degeneration ranging from mild to marked (mean grade 3.3) versus the minimal to mild changes in the 5.8 Gy group (mean grade 1.7). Females showed the same dose-dependent lung damage as males. In the 6.5 Gy group, females had more severe alveolar septal degeneration (mild to moderate, mean grade 2.5) than the 5.8 Gy group (mild, mean grade 2). This trend was also seen with alveolar edema, where the 6.5 Gy group had higher mean grades across all subsets. Notably, inflammatory cellular infiltration was not a significant factor, with only one isolated case showing minimal change in the 6.5 Gy non-surviving female cohort. However, the significance of this finding was uncertain and could have been incidental.

#### Cardiovascular system/heart

**Fig. 8:**
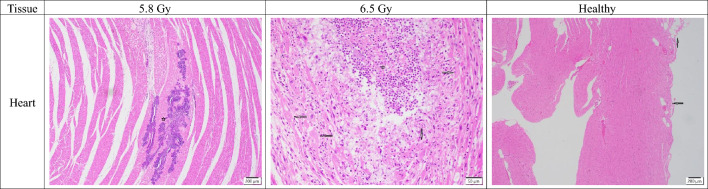
Comparison of heart tissue sections collected from male and female rhesus macaques exposed to 5.8 or 6.5 Gy, as well as healthy tissue. 5.8 Gy (NHP ID RA1272M, scale bar = 200 μm) There is an extensive area of bacterial colonies (star), however there are no corresponding inflammatory cells or cardiac necrosis; 6.5 Gy (NHP ID RA1493F, scale bar = 50 μm) There is mild muscle necrosis and loss (star) replaced by polymorphonuclear cells and surrounding necrotic cardiocytes with mild loss of cellular details (arrows); Healthy (scale bar = 200 μm) Heart is essentially normal, there are occasional lipid cell accumulation on the epicardial layer as part of normal structure (arrows).

All evaluated heart tissues showed minimal to no histopathological changes, with no discernible cardiocyte degeneration in any of the subjects. While cardiocyte necrosis was minimal overall, an unexpected finding was observed in the non-surviving males, where the 6.5 Gy group had no necrosis (mean grade 0), whereas the 5.8 Gy group showed a slight presence (mean grade 0.33). In contrast, non-surviving females followed the expected trend, with the 6.5 Gy group showing a minimal increase in necrosis (mean grade 0.5) compared to the 5.8 Gy group, which showed none. Inflammatory cellular infiltration was also generally absent, with the only exception being a minimal increase in the 6.5 Gy non-surviving females (mean grade 0.5) (Fig. 8). Lastly, bacterial infiltration was identified in one male 5.8 Gy non-surviving subject (RA1272M) and one male 6.5 Gy non-surviving subject (RA2693M), with the 5.8 Gy male showing more focally extensive involvement (grade 2 vs. grade 1).

### Vital signs and clinical parameters

Throughout the 60-day study, animals in the 5.8 and 6.5 Gy groups underwent regular vital sign assessments to monitor their physiological responses to TBI. Tracking these measures allowed us to evaluate the systemic effects of radiation exposure. The following parameters were measured: body temperature (°C), heart rate, weight, weight percent change, systolic blood pressure (BP), diastolic BP, and arterial mean. Overall, these assessments, which are shown in Supplementary Fig. 2, demonstrated that both cohorts maintained stable values in temperature, heart rate, and weight, with only minor fluctuations in weight percent change, systolic BP, diastolic BP, and arterial mean over time. No significant group differences were found in any of the parameters when comparing each radiation dose group at each time point.

### CBC

**Fig. 9:**
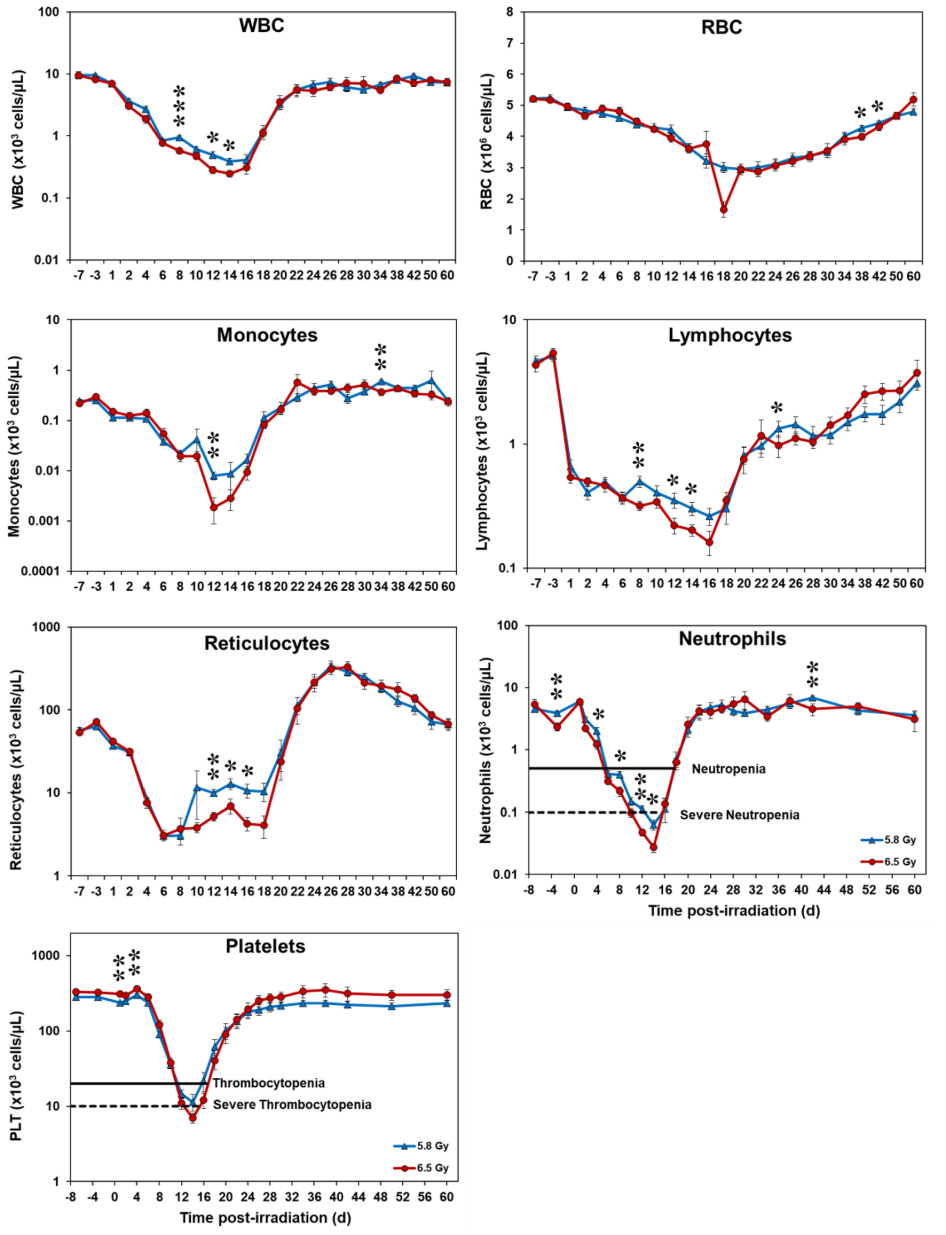
Complete blood counts of male and female rhesus macaques exposed to 5.8 and 6.5 Gy TBI. The data for each time point is presented as the mean for each group. A significant difference between the groups is denoted by the number of asterisks where *, **, and *** correspond to *p* < 0.05, 0.01, and 0.001, respectively. This statistical approach was performed using a one-way ANOVA to compare the two radiation doses at each time point.

Evaluating the CBC profiles was critical for understanding the hematopoietic response to TBI and how blood profiles were impacted by the two radiation doses measured. The following seven parameters were found to be of importance, including WBCs, RBCs, monocytes, lymphocytes, reticulocytes, neutrophils, and platelets, which can be found in Fig. 9. Sex-stratified comparisons for each radiation dose group across these parameters are shown in Supplementary Fig. 3. Trends between males and females were generally comparable throughout the study, with only modest, parameter-specific differences observed at select time points, and no consistent sex-dependent pattern across endpoints. The findings revealed that all cell lineages were significantly affected, but their unique patterns of cellular decline and subsequent recovery, as well as the severity of their suppression, varied between 5.8 and 6.5 Gy. The remaining four parameters, HGB, HCT, basophils, and eosinophils, can be found in Supplementary Fig. 4.

Following irradiation, WBC counts dropped significantly, decreasing more than 28-fold from day 1 to their nadir on day 14 (*p* = 0.017). Recovery began quickly thereafter, with levels rising by day 16 and approaching baseline as early as day 26. By day 60, WBC counts had stabilized to just below baseline in both groups. In contrast, RBC counts declined more gradually and over a prolonged period. In the 6.5 Gy group, RBCs decreased by less than 3.5-fold, reaching a notable drop on day 18. Overall, RBC recovery was slower, with the 6.5 Gy group returning to baseline by day 60, whereas the 5.8 Gy group remained slightly below baseline.

Monocytes exhibited a biphasic temporal response. At the day 12 nadir, the 6.5 Gy group showed significantly greater suppression (*p* = 0.004) but subsequently recovered to surpass baseline levels by day 60, while the 5.8 Gy group remained slightly below baseline at the study’s end. Lymphocyte counts dropped rapidly within day 1 post-irradiation, with both groups reaching a nadir on day 16. Between days 6–16, the 6.5 Gy group had substantially lower lymphocyte counts than the 5.8 Gy group, with significant group differences on day 8 (*p* = 0.005), day 12 (*p* = 0.039), and day 14 (*p* = 0.024). Although recovery began shortly thereafter, both groups showed incomplete recovery, remaining just below baseline by day 60.

Reticulocyte levels, a marker for bone marrow RBC production, dropped sharply in both groups after day 2 post-irradiation. The 5.8 Gy group exhibited an increased recovery between days 8–18, with significantly higher levels observed at several time points compared to the 6.5 Gy group, which showed a slower, more gradual increase over this same period. Between days 18–28, both groups experienced a substantial surge in reticulocyte levels. By day 60, counts had moderately decreased but remained above baseline, suggesting a sustained restoration of erythropoietic function.

Neutrophil counts declined significantly from day 1 post-irradiation, with the 6.5 Gy group remaining in severe neutropenia longer than the 5.8 Gy group. Both groups reached a nadir on day 14 (*p* = 0.024). Recovery began thereafter, although significant differences persisted during the neutropenia phase (day 12; *p* = 0.001) and the recovery phase (day 42; *p* = 0.002). By the end of the study, neutrophil counts had risen to just below baseline in both groups. Platelet counts declined significantly beginning on day 4 (*p* = 0.002), with the 6.5 Gy group experiencing severe thrombocytopenia between days 13–15, while the 5.8 Gy group did not reach this level of severity. Recovery, similar to that observed in neutrophils, began after day 14. By day 60, platelet counts had returned to just below baseline in both groups, with the 6.5 Gy group ultimately surpassing the 5.8 Gy group in restoration levels. The CBC parameters described above, along with their key findings, are summarized in Table 3.

**Table 3:** Overview of longitudinal CBC responses to total-body irradiation, comparing hematologic suppression and recovery patterns following 5.8 and 6.5 Gy exposure.

Parameter	Initial decline	Nadir (timing)	Dose-dependent differences	Recovery pattern	Status at day 60
WBCs	Rapid (day 1); > 28-fold decrease	Day 14	Similar suppression magnitude	Rapid recovery beginning day 16	Slightly below baseline (both doses)
RBCs	Gradual, prolonged decline	Day 18 (6.5 Gy)	Greater suppression at 6.5 Gy; < 3.5-fold decrease	Slow recovery	6.5 Gy returned to baseline; 5.8 Gy slightly below
Monocytes	Biphasic decline	Day 12	6.5 Gy showed significantly greater suppression	Robust rebound	6.5 Gy above baseline; 5.8 Gy slightly below
Lymphocytes	Rapid (day 1)	Day 16	6.5 Gy significantly lower between days 6–16	Partial recovery	Incomplete recovery; slightly below baseline (both doses)
Reticulocytes	Sharp drop after day 2	Day 6	Faster recovery at 5.8 Gy (days 8–18)	Strong surge between days 18–28	Above baseline (both groups); sustained erythropoiesis
Neutrophils	Rapid (day 1)	Day 14	Prolonged severe neutropenia at 6.5 Gy	Rapid recovery after day 14	Slightly below baseline (both doses)
Platelets	Sharp decline beginning on day 4	Days 13–15	Severe thrombocytopenia only at 6.5 Gy	Rapid recovery after day 14	Slightly below baseline; higher recovery at 6.5 Gy

CBC parameters with minimal dose-dependent variation (HGB, HCT, basophils, and eosinophils) were excluded from this analysis.

Surviving animals in the 5.8 Gy group were compared to 6.5 Gy surviving animals using linear mixed-effects models to assess significant longitudinal changes over time while accounting for repeated measures within each animal (Supplementary Tables 8–9). WBCs, platelets, neutrophils, lymphocytes, eosinophils, and basophils demonstrated a significant effect of time only, suggesting no evidence of differential dose-dependent trajectories. In contrast, RBCs, hemoglobin, hematocrit, monocytes, and reticulocytes had significant dose × time interactions, suggesting these patterns both changed over time and followed different trajectories between doses. Non-surviving animals between 5.8 and 6.5 Gy were also compared over time up to day 16 using a similar method (Supplementary Tables 10–11). WBCs, platelets, neutrophils, lymphocytes, eosinophils, and basophils demonstrated significant time effects with no significant interactions between dose and time, while RBCs, hemoglobin, hematocrit, monocytes, and reticulocytes showed significant time × dose interactions. Time point-specific comparisons were then performed between radiation dose groups using estimated marginal means. After FDR corrections, only a few isolated instances of significance were observed in either comparison, suggesting no consistent dose-dependent separation over time.

### Serum biochemistry

Blood samples for serum biochemistry analyses were collected pre- and post-irradiation at six selected time points throughout the 60-day study. Chemistry analysis revealed that serum markers showed significant alterations following TBI, including changes in renal (creatinine, uric acid), hepatic metabolites (total bilirubin, cholesterol, direct HDLC), nonspecific tissue injury (LDH), and pancreatic (lipase) parameters (Supplementary Fig. 5). Key findings for the clinical chemistry parameters described here are summarized in Table 4. In brief, serum creatinine levels were significantly lower in the 6.5 Gy group compared to the 5.8 Gy group across all pre- and post-irradiation time points, with differences persisting through day 60. The 6.5 Gy group had significantly higher uric acid concentrations pre-irradiation compared with the 5.8 Gy group (*p* = 0.026). By day 2, post-TBI, uric acid concentrations in the 6.5 Gy group were markedly lower than those in the 5.8 Gy group (*p* = 0.002), reflecting a drastic reduction following irradiation. Midway through the study, however, uric acid concentrations in the higher-dose group increased and surpassed levels of the 5.8 Gy group by the end of the study.

**Table 4:** Overview of longitudinal serum biochemistry responses to total-body irradiation, comparing dose-dependent perturbations and recovery patterns following 5.8 and 6.5 Gy exposure.

Functional category	Parameter	Dose-dependent differences	Recovery pattern	Status at day 60
Renal	Creatinine	Consistently lower levels at 6.5 Gy compared to 5.8 Gy across all timepoints	Early decrease with minimal recovery over time	Remained below baseline; persistently lower at 6.5 Gy
	Uric acid	Higher pre-irradiation levels at 6.5 Gy with greater early suppression; late rebound exceeding 5.8 Gy	Sharp post-TBI decrease followed by mid- to late-study rebound	Near or above baseline; higher at 6.5 Gy
Hepatic metabolites	Total bilirubin	More pronounced and sustained reduction at 6.5 Gy	Early decline with limited recovery	Below baseline, especially at 6.5 Gy
	Cholesterol	Greater mid-study reduction at 6.5 Gy	Transient decrease followed by recovery after day 28	Near baseline; 6.5 Gy slightly higher than 5.8 Gy
	Direct HDLC	Greater suppression across time at 6.5 Gy	Decline with incomplete recovery	Remained significantly below baseline at 6.5 Gy
Tissue Injury	LDH	Greater acute post-irradiation increase at 6.5 Gy	Early peak followed by gradual decline	6.5 Gy above baseline but 5.8 Gy slightly below baseline
Pancreatic	Lipase	Delayed, dose-dependent divergence with higher levels at 6.5 Gy	Gradual increase over time	6.5 Gy elevated above baseline but 5.8 Gy slightly below baseline

Biochemical parameters exhibiting minimal or no dose-dependent variation (albumin, total protein, GGT, chloride, calcium, phosphorus, sodium, potassium, amylase, glucose, BUN, AST, ALT, ALKP, triglycerides, and CO_2_) were excluded from this analysis.

Total bilirubin levels exhibited a consistent decrease throughout the study for the 6.5 Gy group, with significant group differences by day 60 (*p* =  < 0.001). On the other hand, cholesterol and direct HDLC experienced a “V-shaped” decline and rebound in both groups following TBI, reaching nadirs at day 28. The magnitude of the decline appeared greater in the 6.5 Gy group, and at day 60, direct HDLC concentrations were significantly lower in the 6.5 Gy group compared with the 5.8 Gy group (*p* = 0.004). The 6.5 Gy group remained slightly above the 5.8 Gy group for cholesterol levels by the study’s end.

LDH exhibited the most pronounced acute response, with a significant early post-irradiation increase on day 2 (*p* = 0.009) in the 6.5 Gy group, consistent with increased nonspecific tissue injury at the higher dose. Levels gradually declined thereafter but remained elevated relative to baseline by day 60 (*p* = 0.043). Lipase showed a delayed, dose-dependent divergence, with progressively higher levels in the 6.5 Gy group at later time points, reaching significance by day 60 (*p* = 0.036). However, the limited collection days for biochemistry analysis restricted the ability to fully capture the temporal dynamics of these changes. The remaining parameters, which can be viewed in Supplementary Figs. 6–7, showed no specific trend of interest as values remained relatively stable or displayed nonsignificant changes across time points and doses.

## Discussion

Exposure to acute, high doses of ionizing radiation presents difficult medical challenges from both diagnostic and therapeutic perspectives^51^. Such exposures produce complex injuries that underlie ARS and are driven by a cascade of biological events, including direct DNA damage, generation of reactive oxygen species (ROS), vascular compromise, and dysregulated inflammatory responses^6^. The cumulative impact of these processes disrupts homeostasis across multiple organ systems, especially those with high cellular turnover, such as the hematopoietic and GI systems^5,52^. Clinical studies of individuals accidentally exposed to lethal or supralethal doses of radiation have been instrumental in defining the responses of these radiosensitive tissues and in establishing the systemic nature of ARS^53–60^. At extremely high, often near-lethal doses, injury can arise concurrently across multiple organ systems, such that protecting a single radiosensitive compartment is insufficient to ensure survival. Instead, outcomes are determined by the cumulative burden of multi-organ dysfunction, which influences both short-term survival and long-term health^59,61^. This complexity makes clinical management particularly challenging, as symptoms often evolve insidiously from days to weeks and typically follow a nonlinear course, making timely intervention and monitoring difficult.

At present, the four agents and their associated biosimilars approved by the FDA are indicated specifically for H-ARS, leaving other critical systems largely unaddressed^19^. Moreover, no prophylactic options currently exist for warfighters, first responders, or civilians to prevent the onset of radiation-induced damage, highlighting a gap between medical need and available preventative therapies. Under the FDA Animal Rule, efficacy testing of candidate MCMs must therefore rely on well-validated animal models that faithfully recapitulate human responses to radiation exposure^18,25,62^.

The rhesus macaque (*Macaca mulatta*) has emerged as the benchmark large-animal model in this context^30,32,63,64^. With high genetic similarity to humans, as well as comparable physiology, hematology, and tissue architecture, rhesus macaques display injury patterns that closely mirror those observed in human disease progression, particularly in relation to ionizing radiation exposure. Their established use has generated robust survival and pathology datasets, dose-responsive standards, and clinically relevant biomarkers, all of which support regulatory acceptance of MCM development^30,65^. Nonetheless, limitations related to availability, cost, and ethical considerations necessitate careful experimental design and maximize the importance of extracting mechanistic insight from each study.

Previous studies from our laboratory have reported histopathology outcomes in male and female rhesus macaques treated with gamma-tocotrienol (GT3) 24 h prior to irradiation at both high doses (12 Gy PBI and TBI) and lower-to-mid-range doses (4 and 5.8 Gy PBI and TBI)^66,67^. Following 12 Gy PBI and TBI, the most severely affected tissues included the lymphohematopoietic and GI systems, as well as the kidneys and lungs. Similarly, at much lower, sublethal or near-lethal ionizing radiation doses (i.e., 4 and 5.8 Gy PBI and TBI), the lymphohematopoietic and GI systems once again exhibited pronounced and dose-dependent injuries, whereas other tissues, including the liver, kidneys, and lungs, exhibited less severe and less persistent damage. Another study from our laboratory examined histopathology in male rhesus and male cynomolgus macaques (*Macaca fascicularis*) exposed to 5.8 and 6.5 Gy TBI^40^. Results showed that injuries in organs with strong regenerative capacity, such as the lymphohematopoietic and GI systems, were generally comparable between the two species, but tended to be more severe in cynomolgus macaques. On the other hand, tissues with limited regenerative capacity, such as the liver, showed similar pathologies across species.

While these findings are informative, the current study extends upon this prior work. Further investigation is needed to better define the temporal and organ-specific progression of injury at near-lethal and severe ionizing radiation exposure levels in both sexes, incorporating vital sign assessments, CBC profiles, and serum biochemical analyses. In the present study, we evaluated the histopathological consequences of two potentially lethal total-body γ-radiation doses (5.8 and 6.5 Gy) in male and female rhesus macaques. These doses mimic exposures expected to cause life-threatening injury in humans during nuclear or radiological incidents. These analyses provide a comprehensive view of ARS and its systemic effects, while reinforcing the relevance of translational animal models and their continued role in developing safe and effective MCMs. Importantly, by elucidating organ-specific pathophysiological mechanisms, this work helps refine critical hematological and clinical markers that can guide efficacy testing under the FDA Animal Rule.

Across all endpoints examined, radiation dose emerged as the primary determinant of injury severity. At 6.5 Gy, animals generally exhibited more extensive and sustained injury than at 5.8 Gy, with the most pronounced effects observed in radiosensitive organs such as the GI tract, lymphohematopoietic tissues, and, possibly, the pulmonary system as well. In terms of the latter, however, we did note here in this study that in non-surviving animals, lung histopathology had a marked difference in severity relative to the level of ionizing radiation exposure and the average degree of alveolar septal degeneration within select animals (i.e., a mean grade of 3 at 6.5 Gy was compared to a mean grade of 2 at 5.8 Gy). Similarly, the GI tract showed a clear dose–response relationship, with the 6.5 Gy dose causing more severe villi loss and crypt disruption in the jejunum and ileum. No additional pathological features accompanied this injury, as damaged epithelial cells are rapidly sloughed from the lamina propria due to the high turnover rate of villus epithelium, which normally persists for only 3–5 days. Accordingly, the observed villus loss is most consistent with impaired epithelial regeneration stemming from radiation-induced depletion of proliferative stem cells within the crypts. In the spleen, non-surviving animals at 6.5 Gy exhibited more pronounced white pulp depletion (mean grade 4.5) than their 5.8 Gy counterparts (mean grade 4), directly linking the higher dose to more severe lymphatic tissue damage. Notably, surviving animals at both doses exhibited minimal splenic pathology, suggesting that preservation or recovery of lymphoid architecture may be a critical determinant of survival.

The tissue-level injuries of the lymphohematopoietic system generally showed correspondence relative to noted CBC response patterns. Animals at 6.5 Gy experienced deeper and more prolonged nadirs in WBCs, monocytes, lymphocytes, reticulocytes, neutrophils, and platelets, with delayed recovery compared to 5.8 Gy. However, more notable patterns emerged during the recovery phase. While the 6.5 Gy group sustained more profound initial damage, some parameters, such as RBCs and monocytes, ultimately rebounded to surpass baseline levels, a recovery not seen so substantially in the 5.8 Gy group. For example, after a precipitous drop, RBC levels in the 6.5 Gy group recovered rapidly to exceed the 5.8 Gy group’s counts by the end of the study. This overshoot may indicate a more robust compensatory response at the higher dose once critical bone marrow recovery thresholds are reached. This finding highlights the complex nature of marrow recovery after severe radiation injury, suggesting that the body’s regenerative capacity may be triggered more intensely by a greater initial insult. Despite paradoxical histopathological findings in a few instances (i.e., in the sternum of select animals), the noted temporal changes in blood profiles for most irradiated animals consistently mirrored the overall severity of radiation exposure.

Serum biochemistry analyses further reinforced these histological and hematological findings, revealing dose-dependent renal, hepatic, and metabolic perturbations in a few parameters. Lower creatinine and bilirubin levels in the 6.5 Gy group pointed to altered renal clearance and hepatic metabolism, while elevated LDH at early time points indicated acute tissue damage. In contrast, vital signs and body weight remained relatively stable in both groups, suggesting that while TBI induced significant cellular- and tissue-level damage, overall homeostasis was largely maintained throughout the 60-day study period. This was an interesting finding as this apparent decoupling of organ-specific injury from overt systemic dysfunction underscores the resilience of compensatory mechanisms during the early post-irradiation period.

Importantly, these dose-dependent injury patterns often intersected with sex-specific differences that were most evident during recovery rather than the acute injury phase. In the lymphohematopoietic system, females showed slightly greater bone marrow depletion than males, a finding that was more pronounced at the lower dose. For instance, in non-surviving animals, the mean sternum depletion for 5.8 Gy females was a grade 5, significantly higher than the grade 4.5 seen in males at the same dose, and even higher than the grade 4 seen in both sexes at 6.5 Gy. This result likely reflects early mortality in the higher-dose group, where animals succumbed to overwhelming systemic injury before the full, delayed pathological effects, such as extensive marrow depletion, could fully manifest. Bone marrow cells within the sternum undergo continuous and rapid proliferation, making it difficult to distinguish surviving irradiated cells from regenerated cells in H&E-stained sections. However, given the consistently greater cellularity observed in surviving animals compared with non-surviving animals, the observed pattern likely reflects the robust regenerative capacity of bone marrow following sublethal injury, rather than true preservation of the initial cellular population. Consistent with this interpretation, CBC analyses revealed more profound and prolonged cytopenias at 6.5 Gy despite less pronounced histologic depletion. When stratified by sex, females tended to reach slightly deeper lymphocyte and platelet nadirs, whereas males exhibited more prolonged neutropenia, suggesting sex-modified lineage sensitivity within an otherwise dose-driven response.

In contrast to the sternum, splenic injury followed a more predictable pattern, with comparable depletion between sexes at matched doses and clear dose-dependent lymphocyte suppression in peripheral blood. In the GI tract, the interplay between dose and sex was more pronounced. At 6.5 Gy, females generally showed more severe damage than males, a trend evident in their higher villi-to-crypt ratios and greater villi loss in the duodenum and ileum. These injuries occurred alongside deeper and more sustained cytopenias, particularly lymphopenia and thrombocytopenia, which may have compromised mucosal immune surveillance and regenerative capacity. This was consistent with a more robust inflammatory response in females, as surviving females at the higher dose showed slightly more inflammation in the large intestine (mean grade 1.33) compared to their male counterparts (mean grade 0.75). Supporting these findings, serum biochemistry analyses revealed dose-dependent perturbations indicative of greater systemic tissue injury at 6.5 Gy, including transient elevations in LDH and alterations in hepatic and metabolic markers. These findings suggest that female animals may have a heightened sensitivity as observed earlier^38,68^ or a different compensatory response to TBI in certain tissues, such as GI and hepatic tissues, particularly at higher radiation doses.

Within the non-surviving groups, the timing of euthanasia did not appear to be a major determinant of the observed histopathological findings. Overall, no clear differences were observed in histopathology scores between animals euthanized at earlier compared to later time points within the same radiation dose group. This likely reflects both the inherent complexity of moribundity determination, where no single constellation of clinical signs defines a uniform endpoint, and the small sample sizes within the non-surviving groups.

The tissue- and lineage-specific injury patterns observed in this study are best interpreted in the context of intrinsic differences in cellular turnover, regenerative capacity, and susceptibility to radiation-induced DNA damage. Tissues enriched in stem and progenitor cells, such as the bone marrow, spleen, and intestinal epithelium, contain rapidly dividing cell populations engaged in frequent mitosis, rendering them particularly vulnerable to ionizing radiation. Consistent with this biology, these compartments exhibited the most severe dose-dependent injury and the clearest separation between surviving and non-surviving animals. Beyond stem cell depletion, specialized hematopoietic lineages further contribute to downstream pathology. Megakaryocytes undergo repeated rounds of DNA replication without cytokinesis (endomitosis) to sustain platelet production, a feature that may increase susceptibility to radiation-induced genomic damage. Impaired thrombopoiesis, reflected by deeper and more prolonged platelet nadirs at 6.5 Gy, likely contributed to compromised hemostatic and endothelial support, providing a mechanistic basis for the vascular dilation and leakage observed in select tissues. Variability in the regenerative capacity of these proliferative compartments may therefore help explain the delayed, sex-modified recovery patterns observed across hematological, lymphoid, and epithelial tissues following radiation exposure.

Collectively, these findings support our hypothesis that biological sex influences the severity and recovery of dose-dependent histopathological injury following near-lethal TBI, while reinforcing radiation dose as the dominant determinant of overall injury burden. Across multiple organ systems, sex-specific effects emerged most clearly during the recovery phase rather than the acute injury phase, suggesting that sex modulates regenerative and compensatory responses rather than initial radiosensitivity alone. This pattern was particularly evident in lymphoid tissues, where splenic depletion was comparable between sexes at matched doses, yet recovery dynamics appeared closely linked to survival. In parallel, sex-modified differences in pulmonary injury severity and hematological rebound further indicate that sex shapes the trajectory and timing of tissue repair following radiation insult.

Other organs, including the liver, kidneys, urinary bladder, and heart, generally showed minimal to mild changes, with less consistent dose-dependent trends compared to the lungs or GI tract. The subtlety of these findings suggests that while such tissues are susceptible to radiation injury, they may be less critical to short-term survival following TBI at these doses, or that the severity of ionizing radiation damage is more difficult to detect and quantify histologically. Taken together, these results underscore that radiation dose and the acuteness of exposure are the primary determinants of injury severity, while tissue-specific radiosensitivity and individual factors, such as sex, introduce important secondary variations that shape the trajectory of tissue damage and recovery across different organ systems.

It is important to acknowledge several limitations of this study. The limited sample size of select study groups and the unequal sex distribution between dose groups may have constrained the ability to fully resolve sex-specific effects. Nevertheless, these results remain valuable, as a previous study using the same radiation doses (5.8 and 6.5 Gy) had a smaller sample size that included only males and therefore lacked any sex-specific comparisons^40^. The 60-day study period captured acute injury and early recovery phases but did not incorporate long-term functional assessments, such as pulmonary performance, GI absorption, or immune competency, restricting the ability to assess the full spectrum of tissue- and organ-specific responses to TBI. Finally, early euthanasia in some animals reduced the amount of longitudinal data available, limiting the ability to track individual recovery dynamics over time. However, taken together, these findings demonstrate that radiation dose and exposure rate are the dominant determinants of injury severity and trajectory, while sex introduces secondary, tissue-specific modifications that may become more pronounced at higher exposures. The heterogeneous nature of tissue responses and variable recovery dynamics underscores the need for holistic, multidimensional assessments in preclinical models. From a translational perspective, these integrated evaluations highlight the importance of considering both dose and sex in guiding the development of MCMs. A comprehensive understanding of dose-, sex-, and tissue-specific injury patterns will be critical for optimizing therapeutic strategies and ensuring their effectiveness across diverse exposure scenarios and populations.

## Supplementary Information

Supplementary Information 1.

Supplementary Information 2.

## Data Availability

All relevant data are within the manuscript.
