# Supplementary material for: Comparative study of pathology of various organs of rhesus macaques exposed to two different doses of acute total-body radiation
Source: Sci Rep. 2026 Apr 25;16:14034. doi: 10.1038/s41598-026-49844-x (PMC13135069; doi:10.1038/s41598-026-49844-x)
Supplement: Supplementary file 1 — Supplementary Information 1. [file 41598_2026_49844_MOESM1_ESM.pptx]

## Slide 1
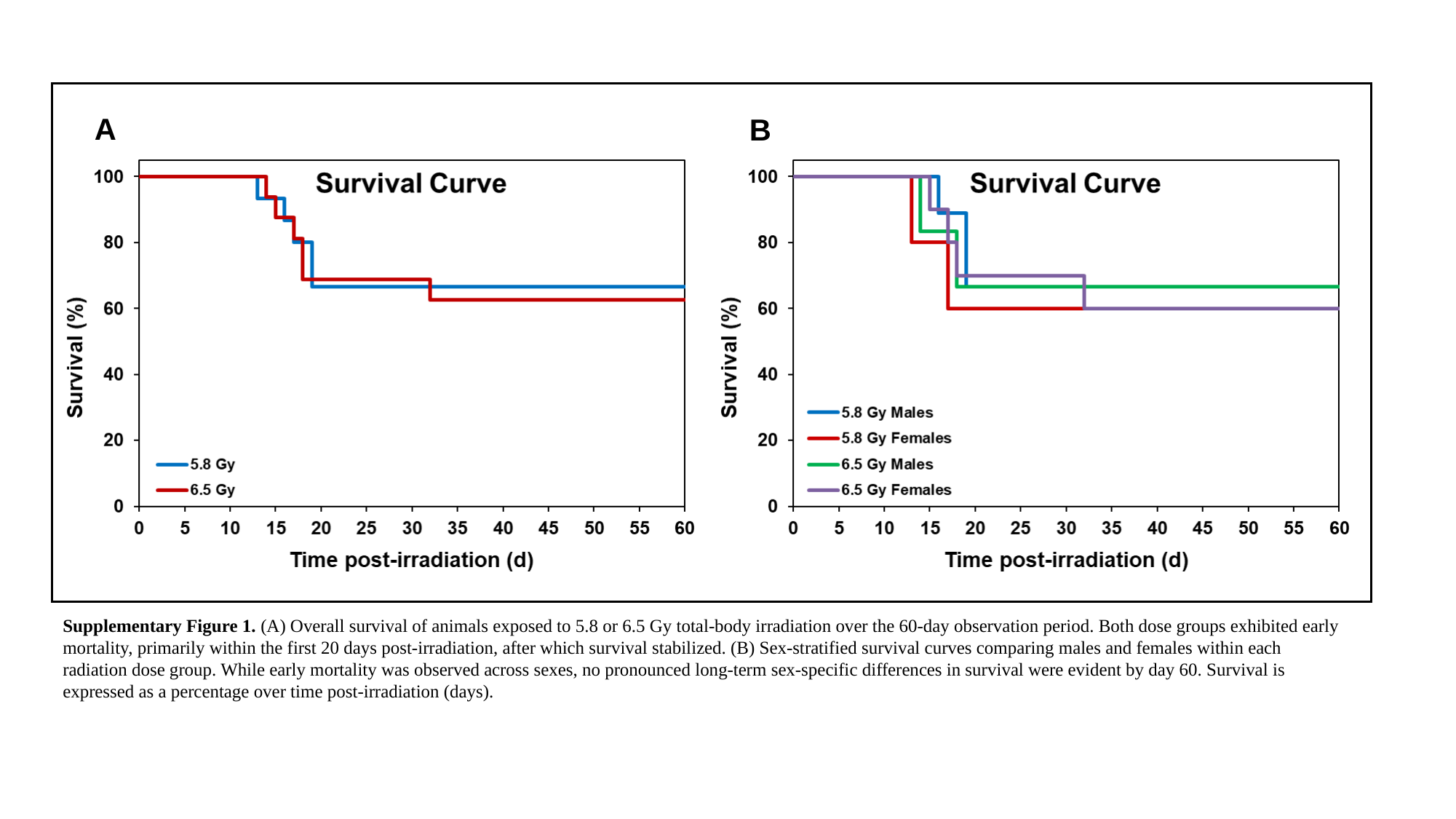

A
B
Supplementary Figure 1. (A) Overall survival of animals exposed to 5.8 or 6.5 Gy total-body irradiation over the 60-day observation period. Both dose groups exhibited early mortality, primarily within the first 20 days post-irradiation, after which survival stabilized. (B) Sex-stratified survival curves comparing males and females within each radiation dose group. While early mortality was observed across sexes, no pronounced long-term sex-specific differences in survival were evident by day 60. Survival is expressed as a percentage over time post-irradiation (days).

## Slide 2
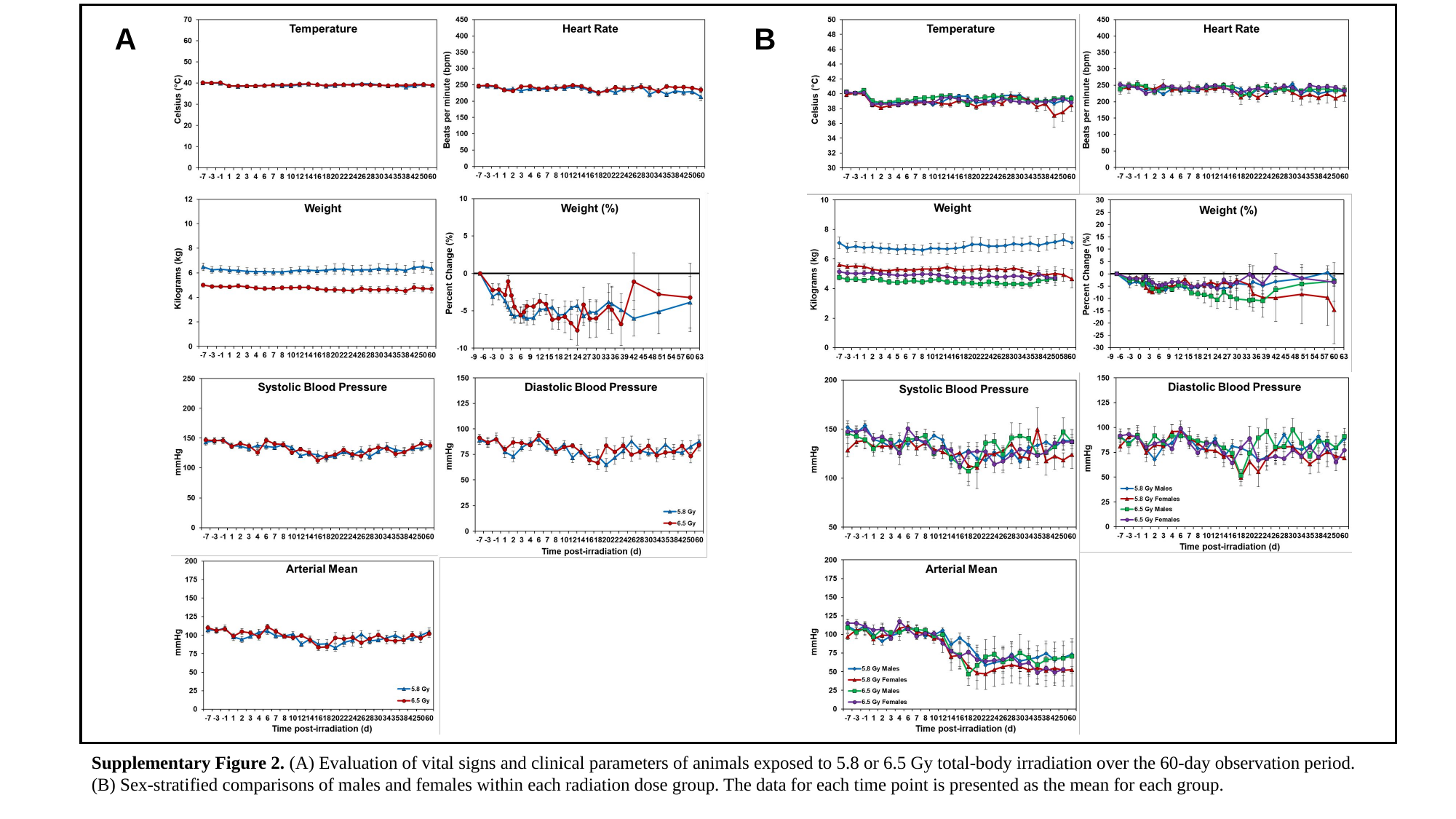

A
B
Supplementary Figure 2. (A) Evaluation of vital signs and clinical parameters of animals exposed to 5.8 or 6.5 Gy total-body irradiation over the 60-day observation period. (B) Sex-stratified comparisons of males and females within each radiation dose group. The data for each time point is presented as the mean for each group.

## Slide 3
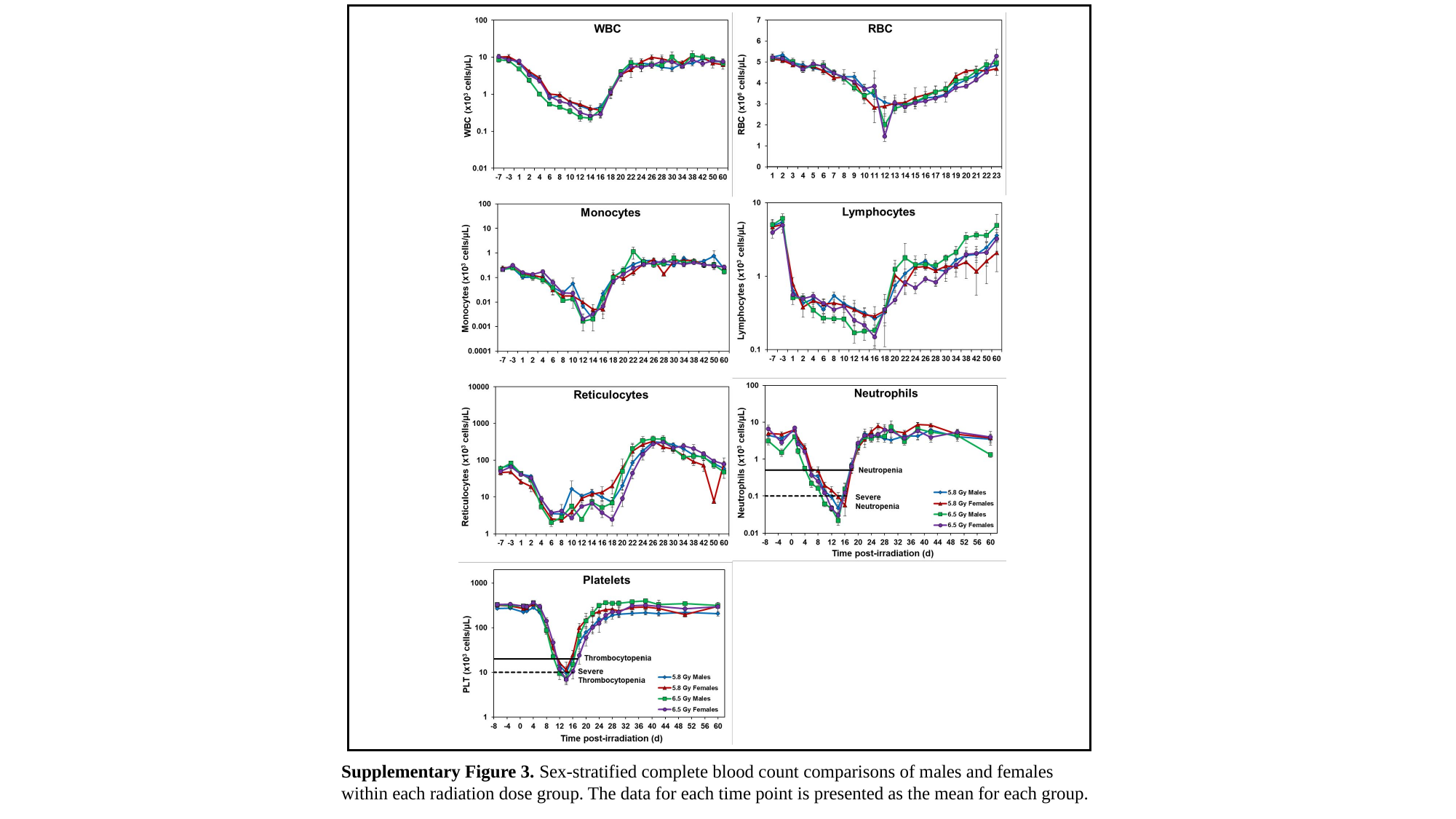

Supplementary Figure 3. Sex-stratified complete blood count comparisons of males and females within each radiation dose group. The data for each time point is presented as the mean for each group.

## Slide 4
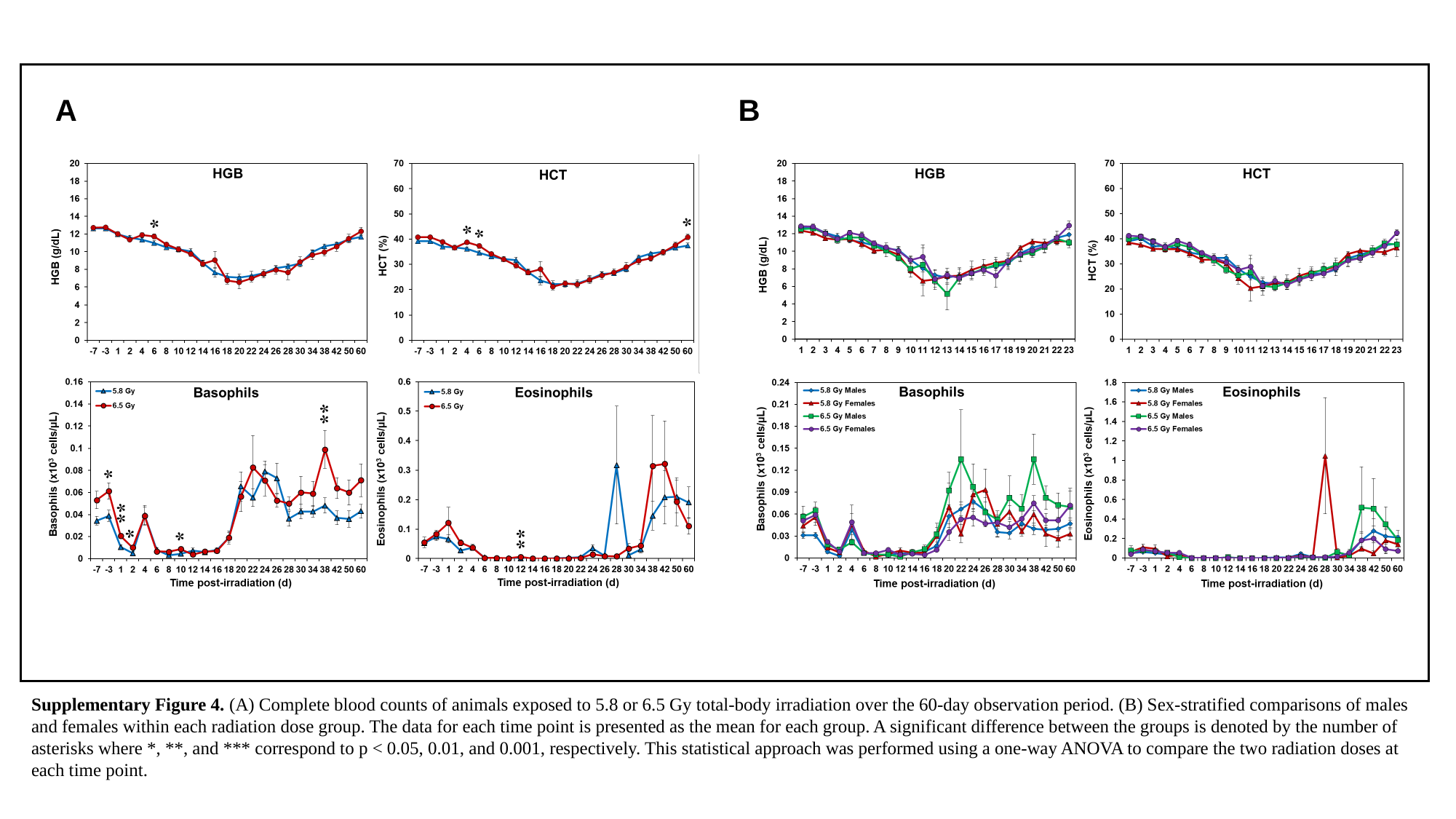

A
B
Supplementary Figure 4. (A) Complete blood counts of animals exposed to 5.8 or 6.5 Gy total-body irradiation over the 60-day observation period. (B) Sex-stratified comparisons of males and females within each radiation dose group. The data for each time point is presented as the mean for each group. A significant difference between the groups is denoted by the number of asterisks where *, **, and *** correspond to p < 0.05, 0.01, and 0.001, respectively. This statistical approach was performed using a one-way ANOVA to compare the two radiation doses at each time point.

## Slide 5
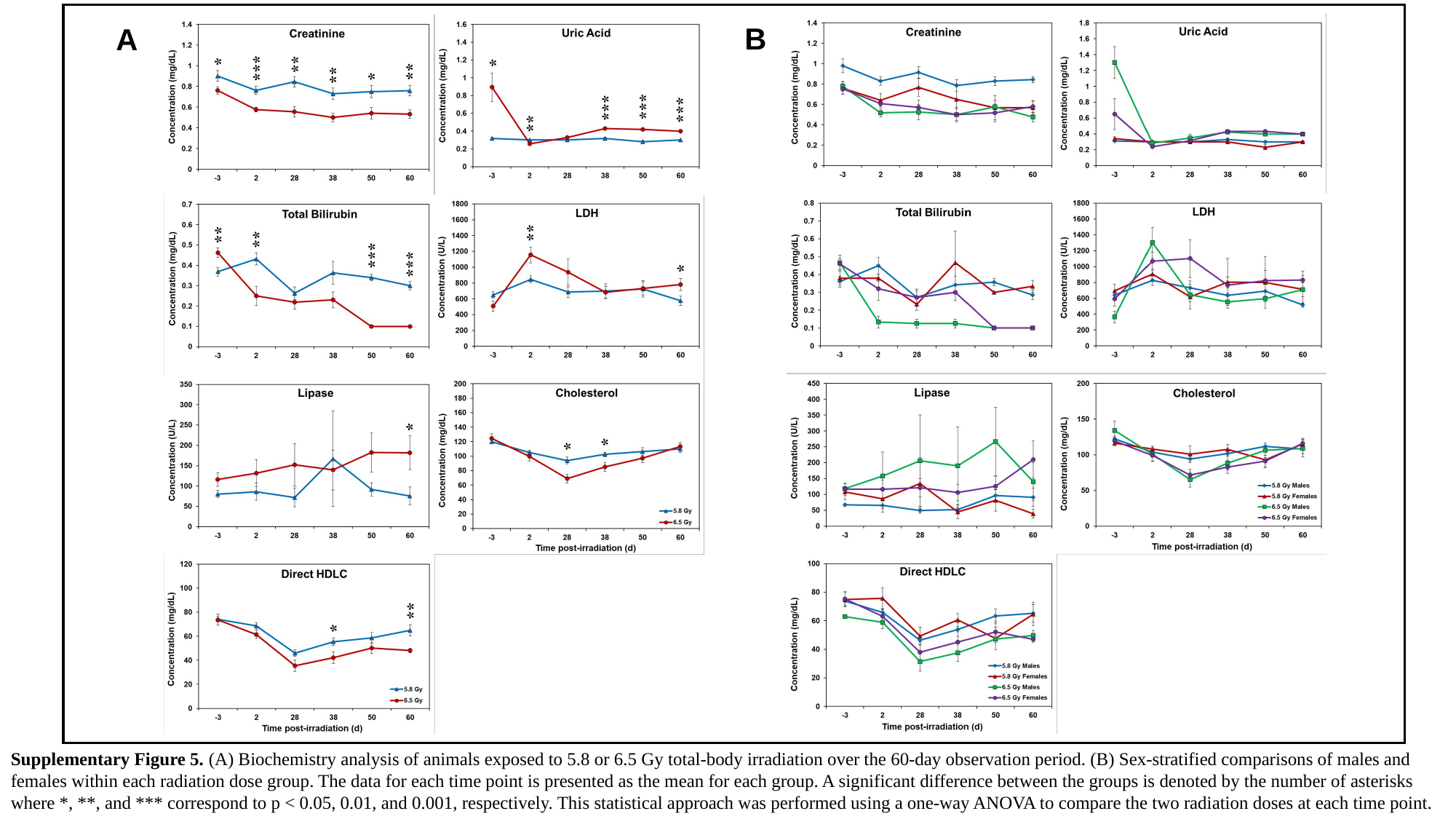

B
A
Supplementary Figure 5. (A) Biochemistry analysis of animals exposed to 5.8 or 6.5 Gy total-body irradiation over the 60-day observation period. (B) Sex-stratified comparisons of males and females within each radiation dose group. The data for each time point is presented as the mean for each group. A significant difference between the groups is denoted by the number of asterisks where *, **, and *** correspond to p < 0.05, 0.01, and 0.001, respectively. This statistical approach was performed using a one-way ANOVA to compare the two radiation doses at each time point.

## Slide 6
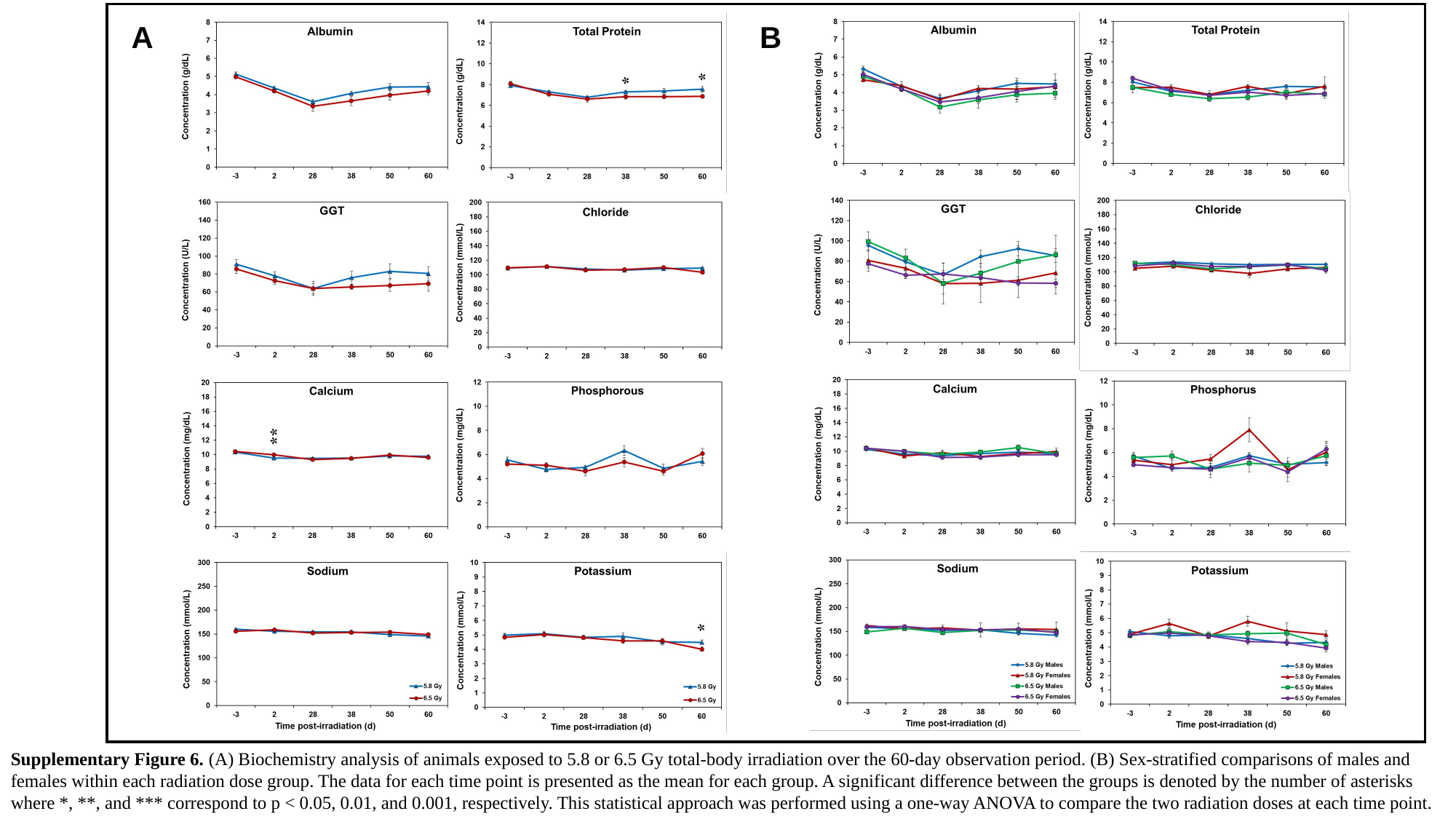

A
B
Supplementary Figure 6. (A) Biochemistry analysis of animals exposed to 5.8 or 6.5 Gy total-body irradiation over the 60-day observation period. (B) Sex-stratified comparisons of males and females within each radiation dose group. The data for each time point is presented as the mean for each group. A significant difference between the groups is denoted by the number of asterisks where *, **, and *** correspond to p < 0.05, 0.01, and 0.001, respectively. This statistical approach was performed using a one-way ANOVA to compare the two radiation doses at each time point.

## Slide 7
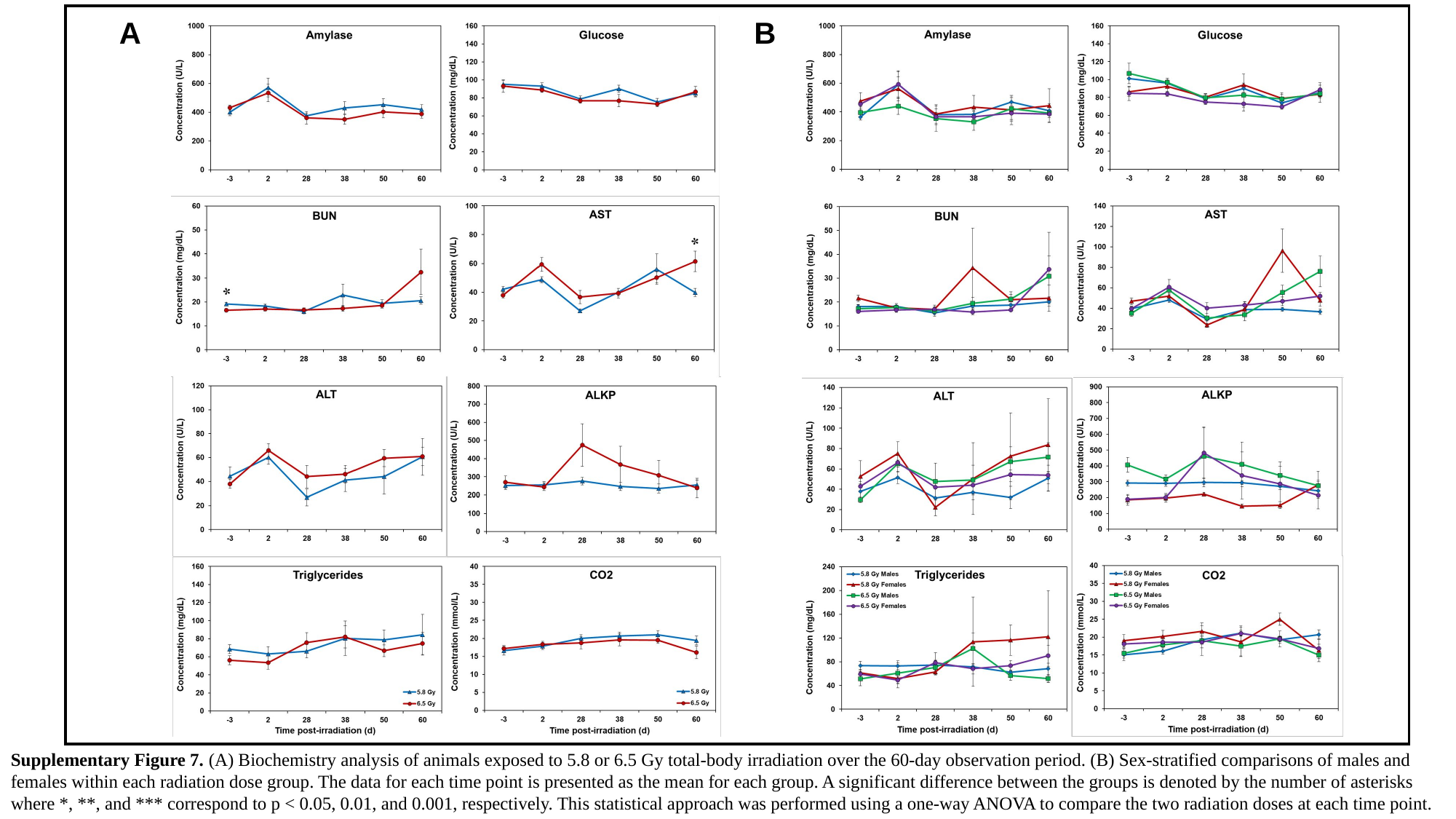

A
B
Supplementary Figure 7. (A) Biochemistry analysis of animals exposed to 5.8 or 6.5 Gy total-body irradiation over the 60-day observation period. (B) Sex-stratified comparisons of males and females within each radiation dose group. The data for each time point is presented as the mean for each group. A significant difference between the groups is denoted by the number of asterisks where *, **, and *** correspond to p < 0.05, 0.01, and 0.001, respectively. This statistical approach was performed using a one-way ANOVA to compare the two radiation doses at each time point.
